# Genetics of cannabis use in opioid use disorder: A genome-wide association and polygenic risk score study

**DOI:** 10.1371/journal.pone.0289059

**Published:** 2023-07-26

**Authors:** Alannah Hillmer, Caroul Chawar, Amel Lamri, Jacqueline Hudson, Flavio Kapczinski, Luciano Minuzzi, David C. Marsh, Lehana Thabane, Andrew D. Paterson, Zainab Samaan

**Affiliations:** 1 Department of Psychiatry and Behavioural Neurosciences, McMaster University, Hamilton, ON, Canada; 2 Department of Medicine, McMaster University, Hamilton, ON, Canada; 3 McMaster University, Hamilton, ON, Canada; 4 Universidade Federal do Rio Grande do Sol, Porto Alegre, Brazil; 5 NOSM University, Sudbury, ON, Canada; 6 Department of Health Research Method, Evidence & Impact, Hamilton, ON, Canada; 7 Program in Genetics and Genome Biology, The Hospital for Sick Children, Divisions of Biostatistics and Epidemiology, Dalla Lana School of Public Health, University of Toronto, Toronto, Ontario, Canada; Yale University School of Medicine, UNITED STATES

## Abstract

**Background:**

Individuals with an Opioid Use Disorder (OUD) have increased rates of cannabis use in comparison to the general population. Research on the short- and long-term impacts of cannabis use in OUD patients has been inconclusive. A genetic component may contribute to cannabis cravings.

**Aims:**

Identify genetic variants associated with cannabis use through Genome-wide Association Study (GWAS) methods and investigate a Polygenic Risk Score (PRS). In addition, we aim to identify any sex differences in effect size for genetic variants reaching or nearing genome-wide significance in the GWAS.

**Methods:**

The study outcomes of interest were: regular cannabis use (yes/no) (n = 2616), heaviness of cannabis use (n = 1293) and cannabis cravings (n = 836). Logistic and linear regressions were preformed, respectively, to test the association between genetic variants and each outcome, regular cannabis use and heaviness of cannabis use. GWAS summary statistics from a recent large meta-GWAS investigating cannabis use disorder were used to conduct PRS’s. Findings are limited to a European ancestry sample.

**Results:**

No genome-wide significant associations were found. Rs1813412 (chromosome 17) for regular cannabis use and rs62378502 (chromosome 5) for heaviness of cannabis use were approaching genome-wide significance. Both these SNPs were nominally significant (p<0.05) within males and females, however sex did not modify the association. The PRS identified statistically significant association with cannabis cravings. The variance explained by all PRSs were less than 1.02x10^-2^.

**Conclusion:**

This study provides promising results in understanding the genetic contribution to cannabis use in individuals living with OUD.

## Introduction

Cannabis use and dependence are estimated to have increased globally over the past two decades, and Cannabis Use Disorder (CUD) is reported as one of the most common drug use disorders [1]. Concerningly, cannabis use is associated with adverse events including impaired ability to concentrate [2, 3], increased anxiety [2, 3], fear or panic [2, 3], as well as possible paranoid feelings and psychosis [2, 3]. In addition, repeated use of cannabis can have long lasting effects including, but not limited to, poor social outcomes (e.g. education outcomes [2, 3], life satisfaction [2], professional and social achievements [2, 3]), physical outcomes (e.g. altered brain development [2, 3], cognitive impairment [2, 3], symptoms of chronic bronchitis [2]) and increased risk of chronic psychologic disorders [2, 3]. Previous studies on cannabis use suggest that neurodevelopmental, pharmacological, metabolic, behavioural, and hormonal differences all contribute to sex differences in cannabis use [4, 5]. Compared to men, women show a greater sensitivity to the subjective effects of cannabis, have faster trajectories to CUD and worse mental health outcomes, such as increased anxiety and a higher risk of early onset psychosis [6, 7]. Given that animal and clinical studies suggest sex-specific differences, it is important to investigate how sex-specific genetic differences are associated with cannabis use outcomes [7]. It has been suggested that a genetic component may exist to cannabis cravings, with heritability estimates from twin studies varying from 41–48% for cannabis use initiation, and from 51–59% for problematic cannabis use in Western countries according to a 2010 meta-analysis [8]. A recent systematic review identifying genetic variants from genome-wide association studies (GWASs) associated with cannabis use found loci of interest near *INTS7*, *PI4K2B*, *CSMD1*, *CST7*, *ACSS1*, and *SCN9A* [9]. Further, to date, five GWAS meta-analyses have been conducted identifying *CADM2*, *ATP2C2*, and *FOXP2* associated with cannabis use, and regions of interest on chromosomes 8 and 10 associated with CUD (lead variants: rs4732724 and rs77300175, respectively) [10–14].

Individuals living with an Opioid Use Disorder (OUD) have higher rates of substance use than the general population, with reports of cannabis use rates of more than 50% [15, 16]. Despite the high prevalence of cannabis use in the OUD population, the short and long-term impacts of cannabis use in OUD are inconclusive, with some studies showing cannabis as a substitute for opioids, some showing no association, and others identifying worse outcomes for patients receiving Medication-Assisted Treatment (MAT) for OUD [17, 18]. Being that cannabis is a widely used substance, with policies supporting the legalization and with a growing viewpoint with the potential substitution of cannabis for other drugs, it is important to understand the genetic factors associated with cannabis use within vulnerable populations, including people suffering from OUD. Further, given known sex differences in human and animal research on cannabis use and its outcomes, there is a lack of studies related to the genetic underpinnings of cannabis use by sex. It is important to understand the sex differences in genetic factors associated with cannabis use.

### Objectives

The study aims to examine if genetic variants are associated with cannabis use within individuals living with an OUD and determine if known genetic variants associated with cannabis use in other populations are associated with cannabis use within individuals living with an OUD through a Polygenic Risk Score (PRS), a method to aggregate the effects of variants across the genome by generating a weighted sum of the number of risk alleles an individual carries [19, 20]. The objectives of the study are to:

1. Conduct a GWAS to identify genetic variants associated with cannabis use
2. Investigate potential sex differences in effect size for any genetic variants approaching or reaching genome-wide significance identified in the previous GWAS
3. Investigate the PRS of CUD and cannabis use with cannabis use traits in this sample

## Methods

This study is reported in accordance to the Strengthening the Reporting of Genetic Association studies (STREGA) guidelines and an accompanying checklist can be found in S1 File [21].

### Study design and setting

**Fig 1:**
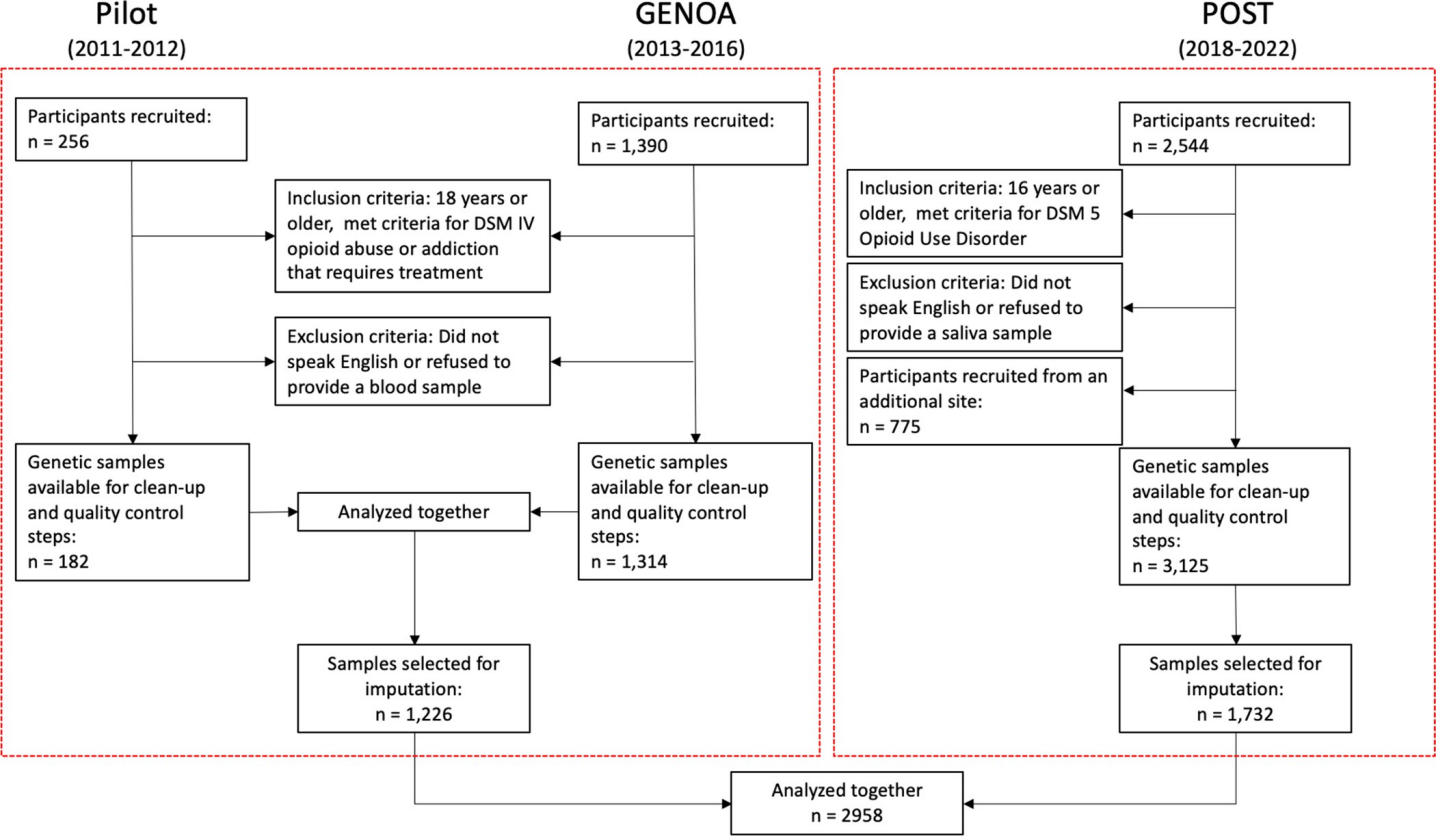
Study diagram.

Data were collected as part of the Genetics of Opioid Addiction (GENOA) pilot, GENOA and Pharmacogenetics of Opioid Substitution Treatment Response (POST) programs, prospective cohort studies designed to identify factors, including genetic risk factors, associated with opioid use and treatment outcomes in patients diagnosed with OUD and receiving treatment. Participants with genotyped data available (n_GENOA PILOT_ = 182, n_GENOA_ = 1314, n_POST_ = 3125) were recruited from 76 Canadian Addiction Treatment Centre (CATC) sites across Ontario, Canada, from 2011–2012, 2013 to 2016 and 2018 to 2022 for the GENOA pilot, GENOA and POST, respectively. The GENOA PILOT and GENOA had identical recruitment, procedures and data clean up steps and thus are henceforth referred to as GENOA. Participants from the POST study included in this analysis were recruited in 2018–2019 due to availability of genotyped data at the time this study was conducted. The POST study included participants recruited from an additional site that was not eligible for this study (n = 775). At study recruitment, participants completed an extensive interview with a trained researcher in which they completed the Maudsley Addiction Profile (MAP), among other measures, and provided a DNA sample [22]. Participants were given a small coffee shop gift card after every face-to-face interview in appreciation for participating. See Fig 1 for a flow diagram of the study cohorts.

The GENOA and POST studies were performed in accordance with the Declaration of Helsinki and were reviewed and approved by the Hamilton Integrated Research Ethics Board (GENOA: 11–056, POST: 4556). Written informed consent was obtained from all participants.

### Participants

For the GENOA study, patients were eligible to participate if they were 18 years or older and met the criteria for Diagnostic and Statistical Manual–fourth edition (DSM-IV) opioid abuse or dependence that required treatment (later replaced in DSM-5 as OUD) [23]. For the POST study, patients were eligible to participate if they were 16 years or older and met the criteria for Diagnostic and Statistical Manual–fifth edition (DSM-5) OUD. The age of participants eligible was lowered to 16 for the POST study to account for younger people entering treatment. Concordance of DSM-IV and DSM-5 criteria has been previously reported, concluding excellent agreement between the two definitions [24]. Patients were excluded from the respective study if they did not speak English, due to lack of resources and validity of instruments in different languages, or declined to provide a blood (GENOA) or saliva (POST) sample (for DNA extraction). In addition to meeting the study eligibility criteria, further inclusion criteria for the present analysis included completing the cannabis related questions on the MAP or self-reported current cannabis use and a viable DNA sample.

### Outcomes and quantitative variables

Outcomes measured in the study include the following:

1. Regular cannabis use, defined as self-reported cannabis use at least twice in a 30-day period.
2. Heaviness of cannabis use, defined as the product of number of days of cannabis use and the typical dose within a 30-day period [25].
3. Cannabis cravings, defined as the score obtained from the Marijuana Cravings Questionnaire–Short Form (MCQ-SF) [26].

Covariates accounted for in the statistical models included: sex, age in years, and three principal components accounting for differences due to population stratification.

### Data sources/measurement

Cannabis use was self-reported during the recruitment interview in which participants indicated the number of days of the past 30 days they used cannabis, the amount used in a typical day and how it was taken (i.e. orally, smoked) as per the MAP.

Regular cannabis use was reported as a binary construct. Participants were reported as regularly using cannabis if they had self-reported cannabis use two or more days in the last 30 days. Heaviness of cannabis use was determined by the number of days of cannabis use and the typical dose used within a 30-day period for self-reported cannabis users. To determine typical dose, we followed a protocol previously used in the literature and converted all amounts to grams of cannabis [25]. In addition, participants who reported one or two “puffs of a joint” were given the equivalent dose as reported in the literature, assuming 10 puffs is equal to 0.5 grams of cannabis [27]. The log of heaviness of cannabis use was used in all statistical analyses to approach a normal distribution.

Post-hoc, we conducted an exploratory analysis using the Marijuana Cravings Questionnaire–Short Form (MCQ-SF) to capture cannabis cravings, reported as a continuous variable with a range of 12–84 based on MCQ-SF score. The MCQ-SF was administered to POST participants who had indicated past month cannabis use. The administration of the MCQ-SF has been previously described [28]. Briefly, the MCQ-SF is a 12-item Questionnaire, scored on a 7-point Likert scale, which assess four components of cannabis cravings [26]. The MCQ-SF components were summed for each participant, and the log of the total score was used for the statistical analysis to approach a normal distribution. The average score for each item on the MCQ-SF can be found in Table S1 in S1 File.

### Quality control checks

Blood samples collected as part of the GENOA study and saliva samples collected as part of the POST study at study recruitment and were genotyped by Génome Québec Innovation Centre using GenomeStudio and the Illumina Global Screening Array– 24 v1.0. Further details on the DNA collection can be found in S2 File. Variant quality control procedures were applied using PLINK v1.90 [29, 30]. Additionally, R version 3.6.3 was used for quality control checks [31].

Samples were excluded if they have a low variant call rate (<99%), inconsistencies between self-reported vs. genetically determined sex or ancestry, or excess heterozygosity suggestive of sample contamination (exchange of DNA between two or more samples). An identity-by-state/decent computation was performed in order to identify and exclude duplicates as well as first and second-degree relatives. Variants were excluded if they have a low call rate across samples (<99%), or if the minor allele frequency was less than 0.05.

Due to the predominantly European ancestry, only data from samples of European decent (n = 2958) were selected for imputation. Imputation was completed by TOPMed Imputation Server (version R2) using the software Eagle v2.4 and Minimac4 for phasing and Single Nucleotide Polymorphism (SNP) imputation, respectively [32–35]. Post-imputation filtering excluded SNPs with Rsq quality metrics of less than 0.3 and minor allele frequencies lower than 0.05. A detailed description is in S1 File.

### Study size

Of the participants enrolled in the GENOA and POST studies, 4621 genetic samples were available. We excluded 775 samples from a cohort that was not eligible to be included in the current study due to different measurements. We further excluded participants based on ancestry as we did not have adequate power to preform subgroup analysis. In total, 2625 participants of European ancestry passed genetic quality control steps and were used for this study. A detailed chart outlining the steps conducted to reach the final sample size is reported in the Figs 8–10 in S1 File.

### Statistical measures

Descriptive statistics were conducted on the total samples to describe the demographic and clinical characteristics, with continuous variables expressed as means with standard deviations, while categorical variables are expressed as counts.

Separate regression analyses were performed to test the association between genetic variants and the outcomes of interest. Logistic regressions were conducted to measure the association of regular cannabis use and linear regressions were used for heaviness of cannabis use and cannabis cravings. Sex, age in years, and three principal components, were adjusted for by using additive models for genotype coding. Further, identical regression analyses were conducted separately for male and female subsets as well the interaction of SNP and sex for SNPs approaching or meeting genome-wide significance.

The statistical software PRSice-2 was used to conduct the PRS to investigate the polygenic risk of cannabis use [36]. Discovery GWAS results were obtained from a large meta-GWAS conducted on CUD which included summary statistics from the Psychiatric Genomics Consortium Substance Use Disorders group, the iPSYCH sample and deCODE sample (downloaded from: https://figshare.com/articles/dataset/sud2020-cud/14842692 on April 18th 2022) and from a GWAS on lifetime cannabis use (downloaded from: https://www.ru.nl/bsi/research/group-pages/substance-use-addiction-food-saf/vm-saf/genetics/international-cannabis-consortium-icc/ on March 20^th^ 2023) [13, 14]. The UCSC*liftOver* tool was used to convert the GWAS summary statistics from GRCh37 to GRCh38 in order to match the target data [37]. Discovery GWAS results were pruned using PRSice-2 using an r^2^ threshold of 0.5 and within 250kb without the use of external reference data. Subset of SNPs were selected at decreasingly liberal P-value thresholds (1, 0.5, 0.4, 0.3, 0.2, 0.1, 0.05, 0.001, 0.0001, 1.0x10^-5^ and 5.0x10^-8^). Using the summary statistics from the GWAS conducted on CUD and lifetime cannabis use previously mentioned, a PRS were calculated and correlations of the PRS and regular cannabis use, heaviness of cannabis use and cannabis cravings were calculated separately (for each outcome and each summary statistics, resulting in 6 PRSs), using the additive model, with all aforementioned covariates adjusted for.

All statistical analyses were performed on PLINK v1.90, R studio 3.6.3 and PRSice-2 [29–31, 36].

## Results

### Participants

Of the 2625 participants whose samples passed the genetic quality control checks, 9 participants with genetic data had missing or incomplete self-reported cannabis use data as collected on the MAP and were excluded from the current study, therefore 2616 participants were included. For the outcome of heaviness of cannabis use, participants were required to self-report at least one day of cannabis use in the past 30 days and report the average number of grams used. Of the 2616 participants, 1321 participants completed the MAP and reported at least 1 day of cannabis use. However, 28 participants did not report the average number of grams of cannabis or were not able to convert their reported usage in grams (e.g. reported the use of cannabis oil), therefore 1293 participants were included in the outcome of heaviness of use. Finally, for the outcome cannabis cravings, of the 1321 participants who reported at least 1 day of cannabis use, 485 individuals did not complete the MCQ-SF and therefore 836 individuals were included in the cannabis cravings analysis. The total number of participants included for each outcome were 2616 for regular cannabis use, 1293 for heaviness of cannabis use and 836 for cannabis cravings. A flow chart is available in Fig S11 in S1 File.

### Descriptive data

For the current study, 965 participants from the GENOA study and 1,651 from the POST study were included. Slightly more participants were male than female (57.45%) with a mean age of 38.23 years (Standard Deviation (SD) = 11.08). The majority of participants had never been married, were unemployed and completed some level of high school. Nearly half of the sample reported regular cannabis use (47.94%), of those who reported cannabis use a mean of 21.30 days out of 30 with and an average of 1.36 grams per day was reported. Cannabis users reported an average score of 37.17 on the MCQ-SF. Finally, 6,377,206 SNPs passed the quality control steps and were included in the GWAS. Up to 381,569 SNPs were included in the regular cannabis use PRS, 381,358 SNPs in the heaviness of use PRS and 380,998 SNPs in the cannabis cravings PRS. Participants demographics can be found in Table 1.

**Table 1:** Sample demographics.

	Total	Male	Female
**N (%)**	2616	1503 (57.45)	1113 (42.55)
GENOA	965	560 (58.03)	405 (41.97)
POST	1651	943 (57.12)	708 (42.88)
**Age in years, Mean (SD)**	38.23 (11.08)	39.89 (11.17)	38.35 (10.90)
**Marital status, N (%)**			
Never Married	1251 (47.83)	761 (50.63)	490 (44.03)
Common law	539 (20.60)	275 (18.30)	264 (23.72)
Currently Married	258 (9.86)	156 (10.38)	102 (9.16)
Separated	241 (9.21)	118 (7.85)	123 (11.05)
Divorced	251 (9.59)	157 (10.45)	94 (8.45)
Widowed	76 (2.91)	36 (2.40)	40 (3.59)
**Currently employed, N (%)**	931 (35.59)	631 (41.98)	300 (26.95)
**Education, N**^**a**^ **(%)**			
Less than grade 9	574 (21.97)	326 (21.69)	248 (22.34)
Grade 9–12	1212 (46.38)	769 (51.16)	443 (39.91)
Trade School	87 (3.33)	59 (4.56)	28 (2.52)
College/University/Graduate School	722 (27.63)	336 (22.36)	386 (34.77)
**Regular use of cannabis, N (%)**	1254 (47.94)	782 (52.03)	472 (42.41)
**Day’s cannabis used in last 30, Mean (SD)** ^**b**^	21.30 (11.59)	21.50 (11.56)	21.05 (11.65)
**Average cannabis dose in g/day, Mean (SD)** ^**b**^	1.36 (2.27)	1.41 (1.69)	1.27 (2.98)
**Log of HOU, Mean (SD)** ^**b**^	1.18 (0.63)	1.21 (0.63)	2.98 (0.62)
**Average MCQ-SF total score, Mean (SD)** ^**c**^	37.17 (16.18)	37.16 (15.87)	37.18 (16.67)
**Log of MCQ**	1.54 (0.19)	1.54 (0.19)	1.54 (0.19)

^a^Data available for n_Total_ = 2,613, n_Male_ = 1503, n_Female_ = 1,110

^b^Data available for n_Total_ = 1,293, n_Male_ = 802, n_Female_ = 491

^c^Data available for n_Total_ = 836, n_Male_ = 507, n_Female_ = 329

### Main results

**Fig 2:**
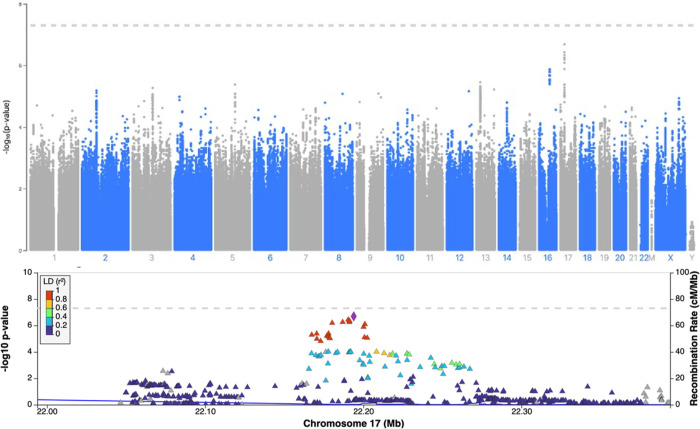
Regular cannabis use Manhattan plot and Regional plot.

**Fig 3:**
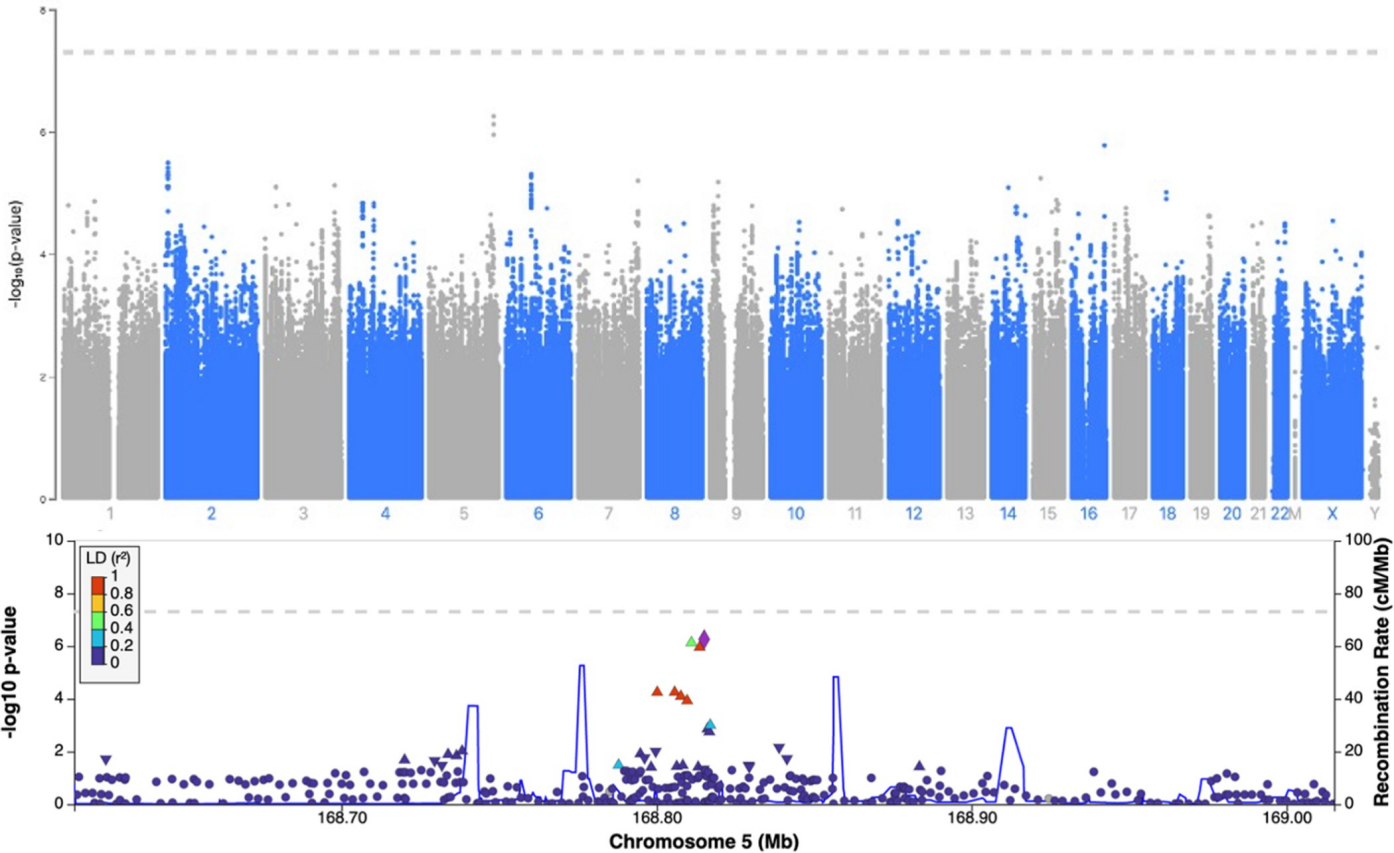
Heaviness of use Manhattan plot and Regional plot.

We identified two lead SNPs in our GWASs approaching genome-wide significance; for regular cannabis use rs1813412 on chromosome 17 (odds ratio (OR) = 1.35, p = 2.05x10^-7^) and for heaviness of use rs62378502 on chromosome 5 (Beta = 0.189, p = 5.56x10^-7^). For both regular and heaviness of cannabis use, a conditional analysis was completed and using the top SNP as a covariate in the analysis did not result in any further significant results, thus no independent variants within the region exist. Manhattan plots and regional plots generated by LocusZoom (using a European reference panel) for the top SNP for regular cannabis use and heaviness of cannabis use can be found in Figs 2 and 3, respectively [38]. Results from the top SNPs of each respective GWAS and QQ plots can be found in the Table S2 and Figs S12 and S13 in S1 File. Results from the PhenoScanner can be found in Table S6 in S1 File, briefly, 6 associations were found for rs1813412 and no associations were found for rs62378502 [39, 40]. Results from the GWAS compared to previous genetic variant associated with cannabis use in the literature can be found in S2 File.

Results from the sex-stratified association analyses between the SNPs approaching GWAS significance for regular cannabis use and heaviness of cannabis use are reported in Table S3 in S1 File, including separate models for males, females and the interaction, and interpreted with the significance threshold of p<0.05. The G allele of rs1813412 was significantly associated with an increased odds of regular cannabis use in both males [OR = 1.31, 95% confidence interval (CI) = 1.13, 1.52, p = 4.32x10^-4^] and females [OR = 1.47, 95% CI = 1.23, 1.76, p = 2.33x10^-5^], however the sex by SNP interaction was not significant (p = 0.23). The C allele of rs62378502 was significantly associated with heaviness of cannabis use in both males [Beta = 0.19, standard error (SE) = 0.05, p = 6.59x10^-5^] and females [Beta = 0.19, SE = 0.07, p = 3.44x10^-3^], however, as with regular cannabis use, the sex by SNP interaction was not significant (p = 0.92).

#### Polygenic risk score results

Results from the PRS include the best-fit model for regular cannabis use, heaviness of cannabis use and cannabis cravings using the CUD GWAS summary statistics are reported in Table 2 and the results for the PRS for the lifetime cannabis use GWAS summary statistics are reported in Table 3. For the PRS models that used the CUD GWAS summary statistics, no PRS model was statistically significant. For the PRS models that used the lifetime cannabis GWAS summary statistics, one PRS model reached statistical significance using the FDR adjusted p-value. Using the p-value threshold of 1.0x10^-5^ for the base data of lifetime cannabis use, the PRS for cannabis cravings was statistically significant with a R^2^ of 1.03x10^-2^ (FDR adjusted p = 0.04). The PRS model fit across p-value thresholds observed in the previously reported GWAS and the bar plots depicting the percentage of variance for regular cannabis use, heaviness of cannabis use and cannabis cravings can be found in Tables S4, S5 and Figs S14-S19 in S1 File, respectively, for both the CUD and lifetime cannabis use summary statistics.

**Table 2:** PRS best model fit for each outcome for CUD.

Outcome	Threshold	PRS R^2^	Full R^2^	Null R^2^	Coefficient	SE	P	Number of SNPs	FDR P-value
**Regular cannabis use**	0.0001	2.50x10^-3^	4.82x10^-2^	4.58x10^-2^	38.6	17.6	**0.03**	204	0.18
**Heaviness of cannabis use**	1.0x10^-5^	9.19x10^-4^	1.51x10^-2^	1.42x10^-2^	-3.7	3.4	0.28	45	0.91
**MCQ-SF**	5.0x10^-8^	1.37x10^-3^	1.56x10^-2^	1.42x10^-2^	0.28	0.3	0.29	2	0.94

MCQ-SF = Marijuana Cravings Questionnaire Short Form, PRS R^2^ = Variance explained by the PRS, Full R^2^ = Variance explained by the full model, Null R^2^ = Variance explained by the covariates, SE = standard error, Number of SNPs = number of SNPs included in the model, FDR = false discovery rate, bolded text = less than 0.05

**Table 3:** PRS best model fit for each outcome for lifetime cannabis use.

Outcome	Threshold	PRS R^2^	Full R^2^	Null R^2^	Coefficient	SE	P	Number of SNPs	FDR P-value
**Regular cannabis use**	1.0x10^-5^	2.90x10^-3^	4.85x10^-2^	4.58x10^-2^	32.80	13.9	**0.02**	74	0.20
**Heaviness of cannabis use**	1	1.85x10^-3^	1.60x10^-2^	1.42x10^-2^	-1072.54	695	0.12	376062	0.48
**MCQ-SF**	1.0x10^-5^	1.03x10^-2^	2.44x10^-2^	1.42x10^-2^	6.77	2.3	**3.39x10** ^**-3**^	74	**0.04**

MCQ-SF = Marijuana Cravings Questionnaire Short Form, PRS R^2^ = Variance explained by the PRS, Full R^2^ = Variance explained by the full model, Null R^2^ = Variance explained by the covariates, SE = standard error, Number of SNPs = number of SNPs included in the model, FDR = false discovery rate, bolded text = less than 0.05

## Discussion

This GWAS did not replicate any previous known genetic associations of cannabis use from the literature. Additionally, the two leading SNPs approaching GWAS significance in this study, rs1813412 on chromosome 17 (not in a gene region) and rs62378502 on chromosome 5 (intron variant on SLIT3, a gene which codes for a protein involved in cell migration) have no known associations with other traits or pathways within the literature by a search of GWAS catalog (May 2022) [41–43]. It is plausible that the lack of replication is due to majority of known GWASs reported in the literature investigated CUD without OUD, a narrow phenotype compared to cannabis use without the associated features of a disorder [9]. Interestingly, there was a significant statistical association between the PRS generated using the summary statistics for lifetimes cannabis use and MCQ-SF, however, the PRS generated using the summary statistics for CUD and the MCQ-SF did not survive multiple correction.

While previous literature on the genetics of cannabis use have identified several SNPs and genes of interest, growing evidence in genetic studies suggest that the majority of genetic variants have small effects which collectively contribute to the risk of certain diseases [9, 20, 44]. We found a statistically significant PRS for cannabis craving, suggesting this score can be applied to assess individual genetic risk of cannabis use. However, the variance explained by genetic variants in all PRS generated were minor, less than 1.03x10^-2^, therefore suggesting that the majority of variance contributing to regular cannabis use, heaviness of cannabis use and cannabis cravings is due to variants not captured by the PRS generated (variants not included in the GWAS summary statistics, low MAF, etc.), non-genetic factors (i.e. environmental) or g*e. From a clinical stance, as genetics are not modifiable as factors such as the environment, there is a greater possibility for changing the modifying risk factors in those who currently use cannabis to reduce vulnerability and increase resilience of cannabis use behaviours [45].

It is important to recognize that this sample includes individuals currently undergoing treatment for an OUD. Individuals who live with an OUD are likely to have used multiple substances and it is relatively common for individuals receiving addiction treatment to engage in continued substance use and polysubstance use while on treatment [15]. Further, in our population it has been previously found that cannabis use is associated with the continued use of other substances while on treatment [46]. However, the evidence surrounding the association between cannabis and illicit opioid use is mixed, with one study reporting cannabis users being less likely to be using heroin and another reporting cannabis use being significantly associated with illicit opioid use in women [25, 46]. Therefore, the results may be obscured due to a shared genetic contribution among various substances, commonalities of addictive substances such as being dopamine-agonists or the high degree of polysubstance use within this population [47].

Finally, this study was unique in stratifying analyses by sex for the top SNP in each GWAS. The current study did not find sex-specific associations for both top SNPs identified in the GWASs, rs1813412 and rs62378502. Nonetheless, investigating sex-specific differences at the genetic level can aid in understanding sex-specific differences at the neurodevelopment, pharmacological, metabolic, and hormonal levels. Although no sex-specific differences were found, it is important to continue to explore sex analyses within the field of genetics to understand sex-specific differences within addiction for clinical implications, ensuring that if underlying genetic differences exist within patterns of substance use, sex-specific treatment decisions are being developed to provide personalized care. Personalized medicine could include informing individuals of their predisposed genetic risk to cannabis use to discourage use based on individual level risk [48].

### Limitations and generalizability

The current study has several limitations. This study utilized self-report data on cannabis use introducing potential reporting bias. Although sensitivity and specificity analyses were previously conducted on self-reported cannabis use behaviour [25], cannabis was an illegal substance at the earlier time of the study (cannabis was legalized in Canada in October 2018), potentially leading to social desirability, responding in a way they believe is appropriate, and recall biases. Bias exists in the measurement of the different components of cannabis, mainly delta-9-tetrahydrocannabinol (THC) and cannabidiol (CBD) which have different effects, as well as in the amount of cannabis used in grams. The amount of THC and CBD included in various strains of cannabis are unable to be measured, and further, there is no standard that currently exists for estimating the amount of cannabis used and methods of consumption [27, 49]. Additionally, missingness within the data in respect to the measure of heaviness of cannabis use, resulting in a smaller sample size for this outcome. Second, due to limited genetic data on individuals of various ancestral backgrounds, the current findings are limited to those of European ancestry. Third, as no other studies to our knowledge have reported on results by sex, they are not comparable to other study findings. Fourth, as our study populations did not screen for CUD we used the MCQ-SF to capture cannabis cravings. Additionally, the GWASs for regular cannabis use and heaviness of cannabis use were both under-powered. Thus, the results of both GWASs and further investigation of sex-specific differences for the top SNPs should be interpreted with caution. Fourth, it is important to note that participation bias in genetic studies exist. While our study collected genetic data at study recruitment mitigating the loss to follow, a bias may exist wherein potential genetic differences in willingness to participate in genetic studies is over-represented [50]. Finally, due to the observational nature of this study, it is not possible to control for all variables or for extraneous confounding variables.

Despite these limitations, the current study provides additional evidence for the genetic liability of cannabis use within a OUD population, a high risk population with polysubstance addiction [51]. While further research is required on the genetic susceptibility to cannabis use within individuals living with an OUD, the current study identifies the need for investigation of the contribution of sex to cannabis use.

## Supporting information

S1 File This file includes additional data clean-up and quality control steps, additional results, regional plots, QQ plots and PRS model of fit pots and the STREGA checklist. (DOCX)

S2 File This file includes a comparison of genetic variants identified in the literature and results for the corresponding variant from the present analysis. (XLSX)
