# Supplementary material for: Genetics of cannabis use in opioid use disorder: A genome-wide association and polygenic risk score study
Source: PLoS One. 2023 Jul 26;18(7):e0289059. doi: 10.1371/journal.pone.0289059 (PMC10370765; doi:10.1371/journal.pone.0289059)
Supplement: S1 File — (DOCX) [file pone.0289059.s001.docx]

Genetics of cannabis use in opioid use disorder: A Genome-Wide Association and Polygenic Risk Score study

Supplementary File 1

**Data clean-up and quality control
Study Descriptions**

The following clean-up and quality control steps were conducted on the Pilot GENOA (n=182), GENOA (n=1,314) and POST data (n=3,125) genetic samples. The Pilot GENOA, GENOA and POST were all prospective cohort studies designed to identify factors associated with opioid use and treatment outcomes including genetic risk factors in patients diagnosed with Opioid Use Disorder and receiving treatment. The Pilot GENOA and GENOA data were merged into and analyzed as one dataset, and are henceforth referred to as the GENOA data (1,2). The POST data included 775 samples from a collaborating site, all of which were excluded at the post-imputation stage as none were of European ancestry. While analyzed separately, the quality control steps taken for the GENOA datasets and the POST dataset pre-imputation were identical. The GENOA datasets and POST data set were merged and analyzed together post-imputation. All analyses were performed on PLINK 1.90 and the RStudio interface of R Version 1.1.453 (3–5).

**Collection of DNA and genotyping**

As part of the GENOA study, whole blood samples were collected. Blood samples were centrifuged, separated and frozen in -20°C within 2 hours of collection at the clinics and then transferred to -80°C freezers located at McMaster University within 1 month of collection. As part of the POST study, approximately 2ml of saliva samples were collected at the baseline using DNAgenotek all-in-one system for the collection, stabilization and transportation of DNA from saliva (OGR-500) (6). DNA was extracted from blood or saliva samples (7) and genotyped by Genomé Quebec using GenomeStudio (v 2.0.4) and the Infinium Global Screening Array – 24 v1.0 (8–10). Illumina released an updated Manifest File for the Global Screening Array – 24 v1.0, resulting in a subset of samples genotyped with Global Screening Array – 24 v1.0 C1 (11). Batch effects were investigated and genetic variant call rates and imputation quality were not impacted.

**Log-transformed Phenotype data**

For the outcome of heaviness of use and cannabis cravings, the log of the raw data was used to approach a normal distribution. The data was transformed sing the RStudio interface of R Version 1.1.453 (3,4) using the log10 of the raw phenotype plus 1. A histogram of the transformed data for each outcome, heaviness of cannabis use and the Marijuana Cravings Questionnaire – Short form (MCQ-SF), is below in Figures S1 and S2, respectively. In addition, the average response for each item on the MCQ-SF can be found in Table S1.


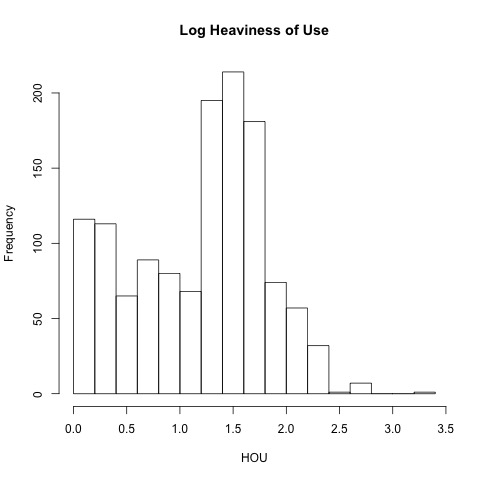


Figure S1. Histogram of the Log of heaviness of use


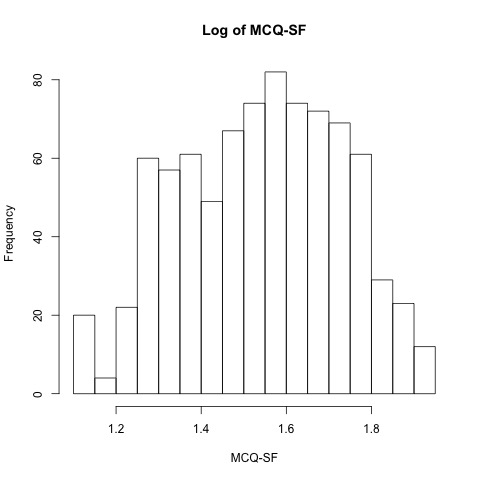


Figure S2. Histogram of the Log of cannabis cravings using the Marijuana Cravings Questionnaire – Short Form

| Table S1. MCQ-SF Questionnaire | |
| --- | --- |
|  | Average (Standard deviation) |
| Using marijuana would be pleasant right now. | 4.74 (2.35) |
| I could not easily limit how much marijuana I used right now. | 1.94 (1.77) |
| Right now, I am making plans to use marijuana. | 2.76 (2.41) |
| I would feel more in control of things right now if I could use marijuana. | 2.10 (1.92) |
| Using marijuana would help me sleep better at night. | 5.82 (1.91) |
| If I used marijuana right now, I would feel less tense. | 3.87 (2.47) |
| I would not be able to control how much marijuana I used if I had some here. | 1.46 (1.32) |
| It would be great to use marijuana right now. | 3.52 (2.49) |
| I would feel less anxious if I used marijuana right now. | 3.29 (2.44) |
| I need to use marijuana now. | 1.59 (1.49) |
| If I were using marijuana right now, I would feel less nervous. | 2.76 (2.31) |
| Using marijuana would make me content. | 3.95 (2.46) |
| **MCQ-SF Total** | 37.80 (16.38) |

**Quality Control**

The genotyped files were converted into .bed, .bim, and .fam files and merged into one dataset and chromosomes that failed genotyping were removed. All samples that were genotyped were cross-referenced with the sample shipment documents to ensure that there were no missing samples.

Missingness per sample and per SNP was estimated using PLINK’s --missing flag. Samples and SNPs with more than 10% (at early QC stages), and 5%, (later in the QC process) were removed.

Four sets of samples were genotyped twice across the Pilot and GENOA data accidently. For each duo of duplicates, the samples with the lowest missingness rates were kept.

Samples with discordant sex information were identified using the --check-sex flag in PLINK. Chromosome X’s inbreeding coefficient was graphed for males and females separately. Males with a coefficient ≥ 0.8 were kept; females with a coefficient ≤ 0.4 were kept.

The sample heterozygosity rates were checked. The resultant values of the heterozygosity rate were calculated using the equation “(N(NM)-O(Hom))/N(NM)” (Number of autosomal genotype observations minus observed number of homozygotes divided by the number of autosomal genotype observations). A histogram was graphed of the heterozygosity rate, and the threshold was determined to be 0.22. Samples with a calculated rate of less than or equal to 0.17 were checked for which ancestry group they belong as lower heterozygosity rates are expected within the certain populations (e.g. Native American ancestry). No samples of less than or equal to 0.17 were removed as all samples were from a Native American ancestry. Heterozygous haploids and nonmale Y chromosome genotype calls were set as missing.


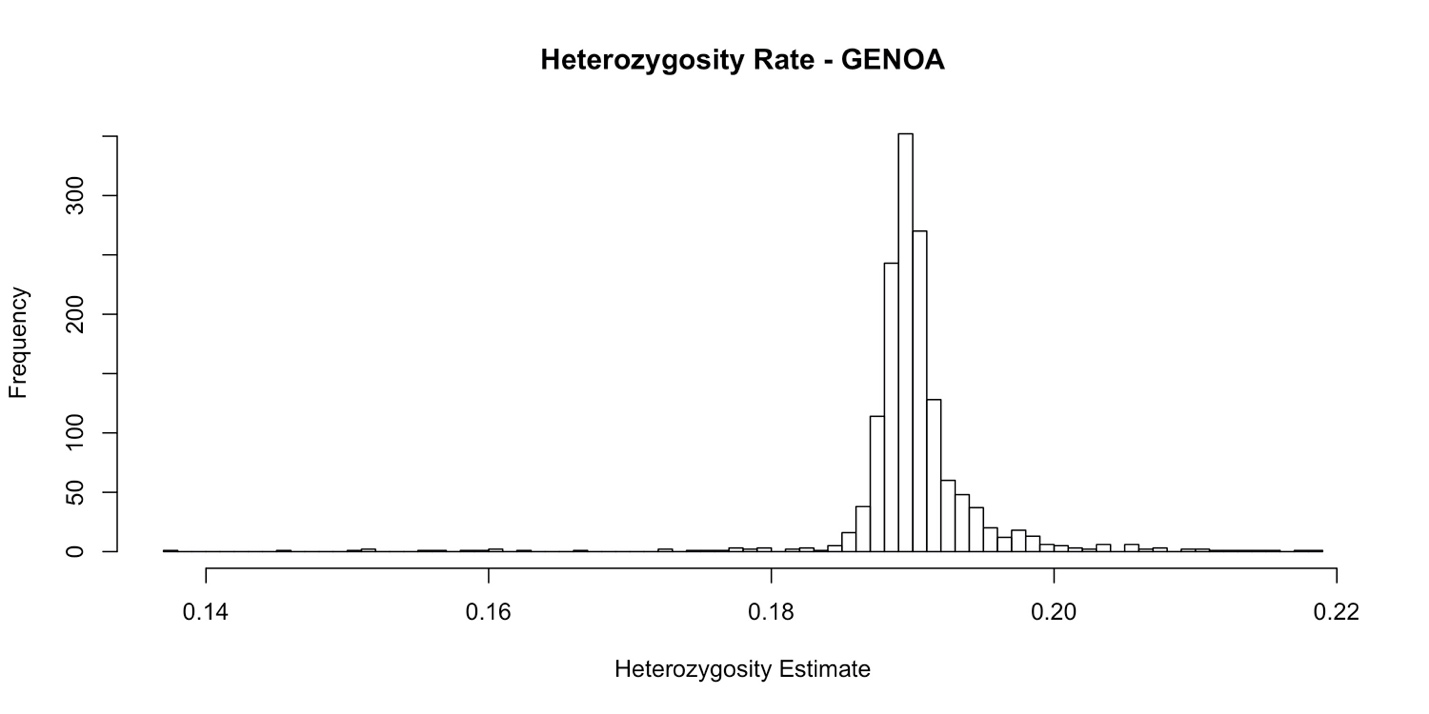


Figure S3. GENOA Heterozygosity Plot


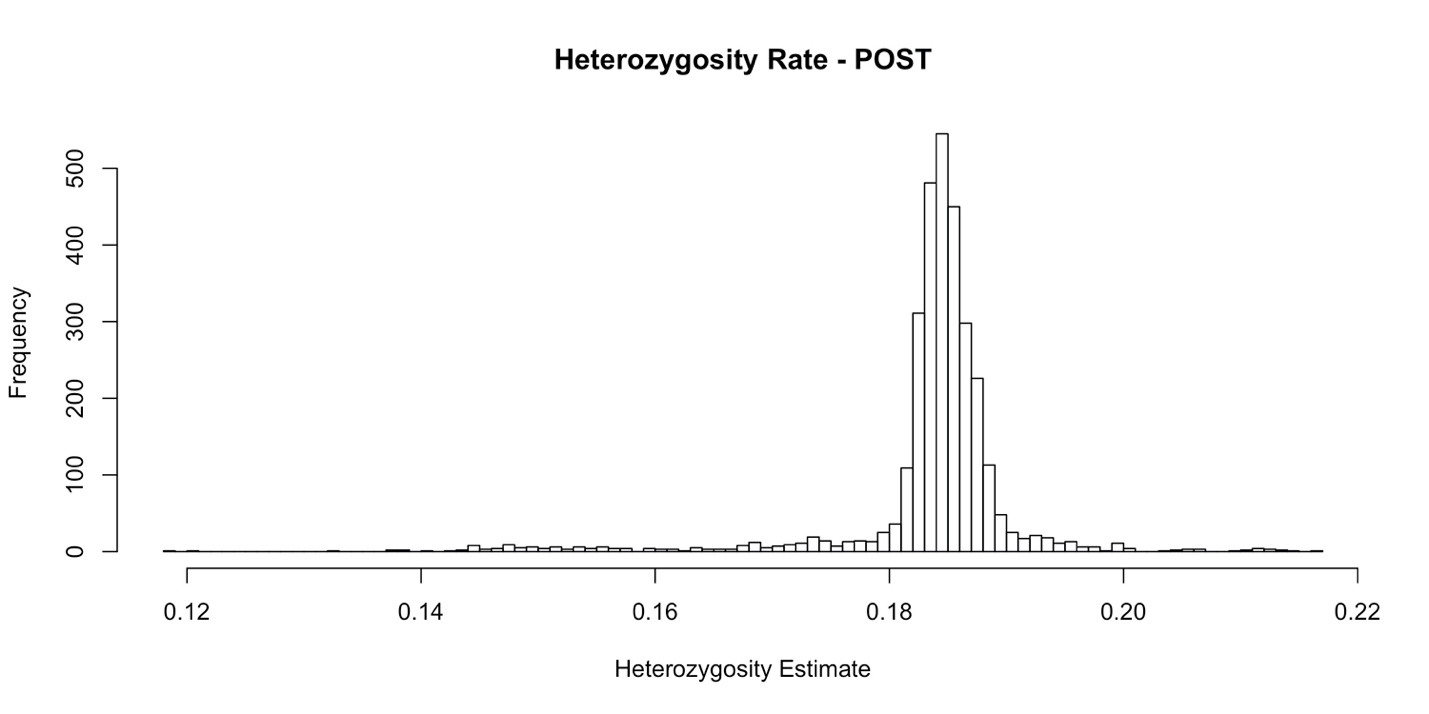


Figure S4. POST Heterozygosity Plot

A principal component analysis was conducted pre-imputation on all samples as part of the ethnicity checks, following the pruning of areas of high range LD (within a 50kb range and r^2 threshold of 0.2). This was conducted in GENOA and POST samples separately, without the use of a reference panel. An additional principial component analysis was conducted at the post-imputation stage and used a reference panel, please see the “Principal Component Analysis” section for further details. The self-reported ancestries of the samples used to colour the points on the principal component scatter plot to visually highlight any outliers. Outliers were defined as samples which did not cluster with other samples of the same self-reported ancestry. Samples whose ancestries were corrected were those that were determined to possibly partially belong to the genetically determined ancestry group (ex. self-reported as 'European' but is 'mixed European and Native North American') by reporting all ancestry groups the sample belongs to. Samples that failed the ethnicity check were removed (n_GENOA_=20, n_POST_=12).

Samples with high relatedness values (PLINK’s --genome output PI_HAT>=0.2) were identified. Along with samples that were believed to be duplicates, have failed the sex check, ethnicity check, and/or genotyping, they were visualized on their respective plate positions to see if any unusual patterns could be observed. Any newly identified duplicates were checked against the case report forms to verify their duplicate status. All verified duplicates were then removed.

**Pre-imputation and imputation**

To prepare the data for imputation by the TOPMed Imputation Server (12), the following steps were run on a Linux operating system. Since at that stage only the European ancestry subset had a sample size large enough for the purpose of our analysis (other ancestries of less than 100 samples would not be powered enough for ancestry-stratified analysis), only samples of European ancestry were submitted for imputation and later analyzed.

The reference alleles for the European ancestry subset were set up to match those from HRC reference panel, follow the steps on the McCarthy Group Tools site (V4.2.11) (13). The frequency file used for the 1000 Genomes Phase 3 match was taken from the McCarthy Group tools (<https://www.well.ox.ac.uk/~wrayner/tools/>) (V4.2.11) (13). SNPs with high MAF (MAF>0.4), differing alleles, not in the reference panel or with an allele frequency difference of >0.2 were removed.

Phasing was done using Eagle2, using TOPMed (14,15).

**Post-imputation filtering and quality control**

The following steps were performed using a virtual machine instance and cloud storage supported by the Google Cloud Platform (<https://console.cloud.google.com/>) (16). Imputed individual chromosome files were recoded from .vcf to .ped/.map files, and then to .bed/.bim/.fam files before being merged into one file on PLINK for easy handling.

The Rsq values were used for filtering. SNPs with equal to or less than 0.3 Rsq were identified to be of low quality and removed. Further, SNPs with MAF<0.05 were removed.

As the GENOA datasets and the POST dataset were merged at this time, duplicates and first and second-degree related individuals were removed, with a prior decision to keep POST samples over GENOA samples due to robustness of the phenotype data within the POST study.

**Other chromosome quality control**

While the X chromosome was included in imputation, other non-autosomal chromosomes were not and thus additional steps were conducted to include them in the analyses. Genetic variants from the follow regions were taken from the raw file, prior to any quality control steps or imputation. For the pseudo-autosomal region of X (XY) and mitochondrial DNA no additional quality control steps were taken. For the Y chromosome, non-pseudo-autosomal regions, females and individuals who failed the sex check (n=2) were removed and sex was not included as a covariate. Finally, for the pseudo-autosomal regions, PAR1 and PAR2, individuals who failed the sex check (n=3) were removed.

**Principal Component Analysis**

To account for population stratification a Principal Component Analysis (PCA) was conducted with data prior to data imputation. Data from the GENOA datasets and POST dataset were combined, and duplicates/related individuals identified post-imputation were removed. Data from the GENOA and POST datasets were merged with the 1000Genome dataset to check for ethnic outliers. The GENOA, POST and 1000Genome datasets were checked for strand flips and corrected. Areas of high linkage disequilibrium and regions of long-range of long-range LD, as reported in the UKBiobank supplementary information Table S13 (17), were removed prior to conducting the PCA. Results from the cleaned PCA, including only European ancestry from the 1000Genome dataset can be found below in Figure S5-7


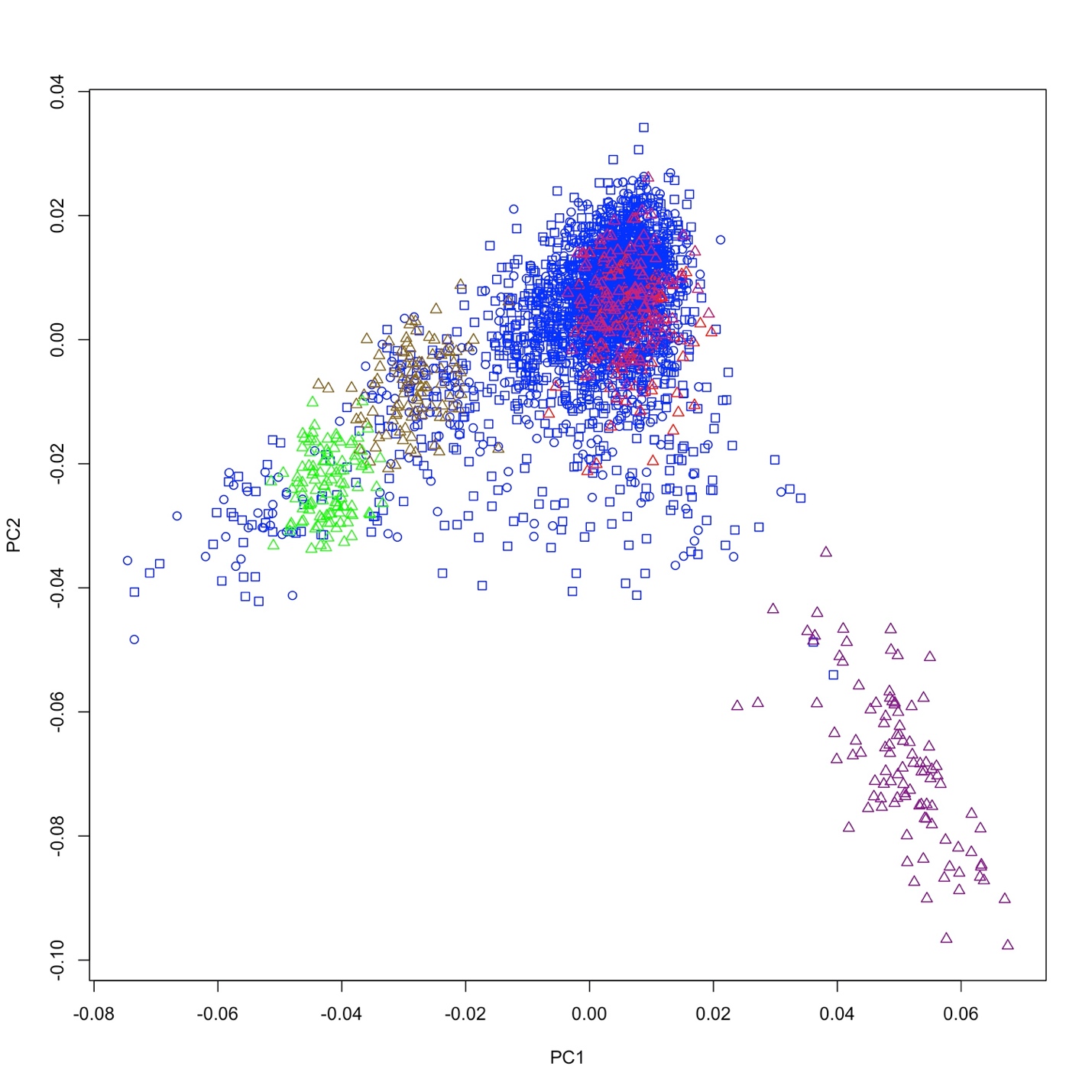


Figure S5. PC1 and PC2 for GENOA, POST and 1000 Genome Data

Legend: Circles = GENOA data, Squares = POST data, Triangles = 1000 Genome data, Blue = European Ancestry as self-reported in the GENOA or POST study, Red = 1000 Genome Utah residents with Northern and Western European Ancestry (CEU), Green = 1000 Genome Toscani in Italia (TSI), Magenta = 1000 Genome Finnish in Finland (FIN), Pink = 1000 Genome British in England and Scotland (GBR), Gold = 1000 Genome Iberian populations in Spain (IBS)


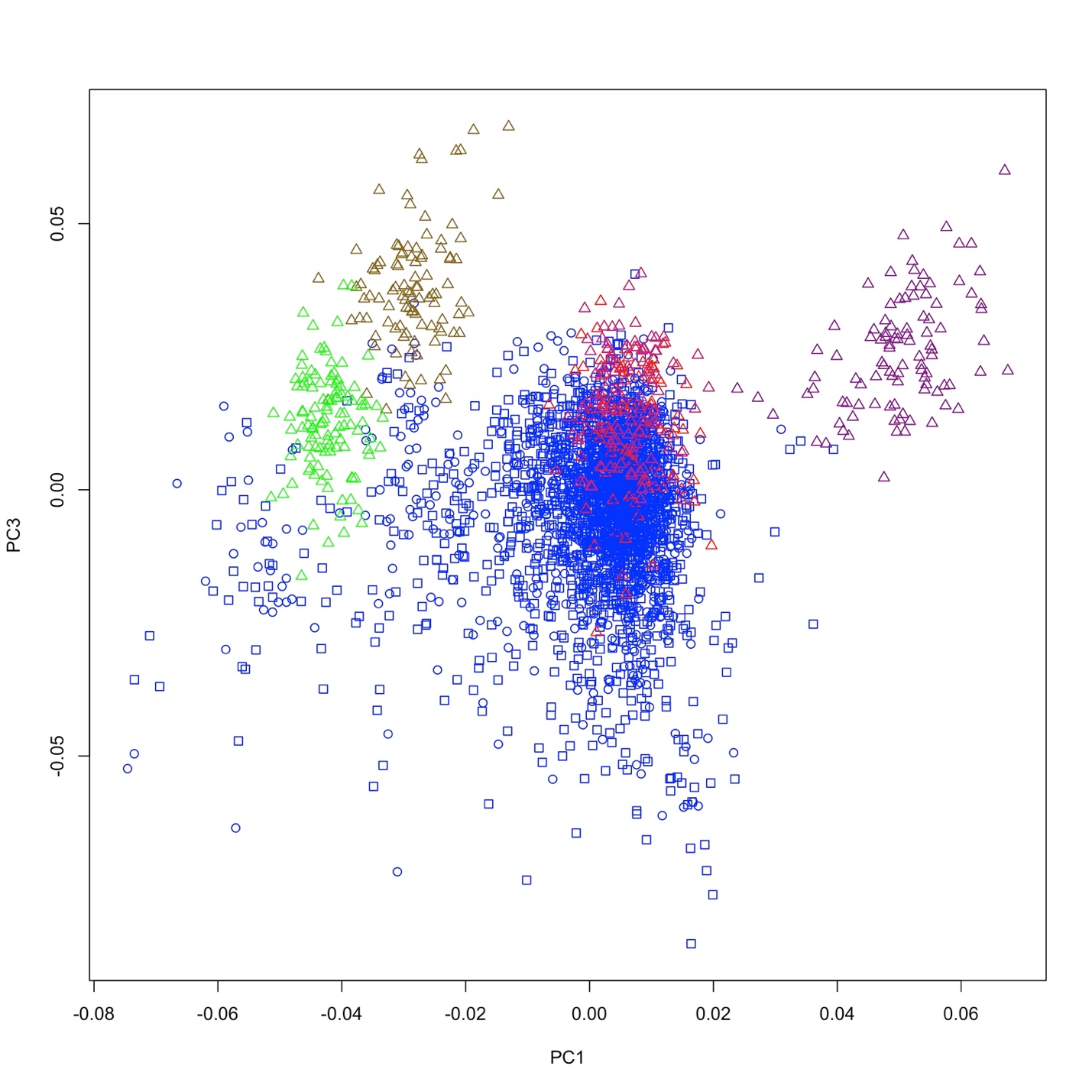


Figure S6. PC1 and PC3 for GENOA, POST and 1000 Genome Data

Legend: Circles = GENOA data, Squares = POST data, Triangles = 1000 Genome data, Blue = European Ancestry as self-reported in the GENOA or POST study, Red = 1000 Genome Utah residents with Northern and Western European Ancestry (CEU), Green = 1000 Genome Toscani in Italia (TSI), Magenta = 1000 Genome Finnish in Finland (FIN), Pink = 1000 Genome British in England and Scotland (GBR), Gold = 1000 Genome Iberian populations in Spain (IBS)


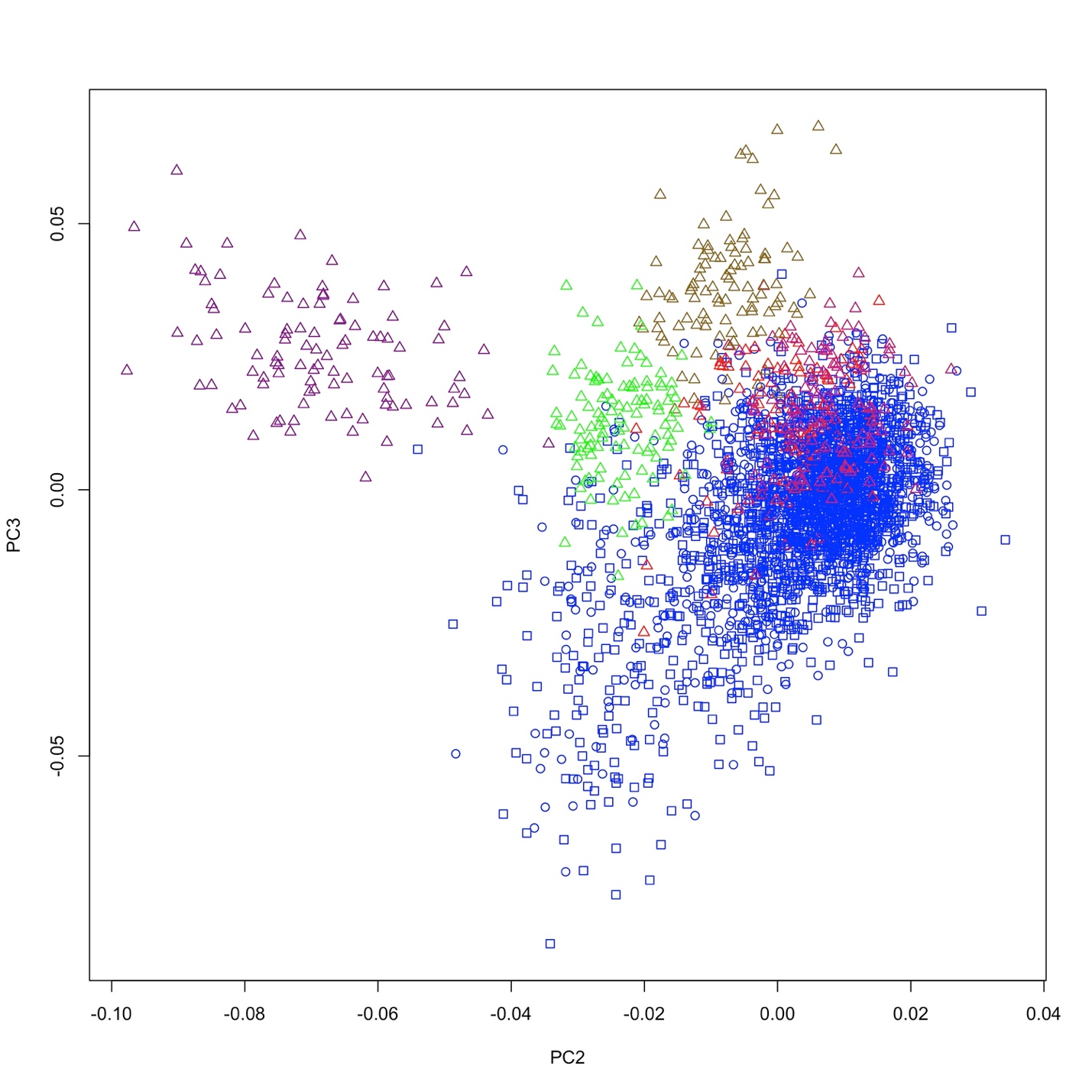


Figure S7. PC2 and PC3 for GENOA, POST and 1000 Genome Data

Legend: Circles = GENOA data, Squares = POST data, Triangles = 1000 Genome data, Blue = European Ancestry as self-reported in the GENOA or POST study, Red = 1000 Genome Utah residents with Northern and Western European Ancestry (CEU), Green = 1000 Genome Toscani in Italia (TSI), Magenta = 1000 Genome Finnish in Finland (FIN), Pink = 1000 Genome British in England and Scotland (GBR), Gold = 1000 Genome Iberian populations in Spain (IBS)

Figure S8. Flow chart of GENOA datasets pre-imputation

Figure S9. Flow chart of POST data pre-imputation

Figure S10. Flow chart of GENOA and POST datasets post-imputation

Figure S11. Flow chart of Participant inclusion for each outcome

| Table S2. Lead SNPs from Regular Cannabis use and Heaviness of Cannabis Use GWAS | | | | | | | | | | |
| --- | --- | --- | --- | --- | --- | --- | --- | --- | --- | --- |
| **Outcome** | **Chr** | **SNP** | **BP (GRCh 38)** | **A1** | **A2** | **MAF** | **OR/BETA** | **95% CI/SE** | **P** | **RSQ** |
| **Regular Cannabis Use** | 17 | rs1813412 | 22193901 | G | A | 0.38 | 1.35 | 1.21, 1.52 | 2.05x10^-7^ | 0.75 |
| **Heaviness of Cannabis Use** | 5 | rs62378502 | 168815119 | A | C | 0.11 | 0.19 | 0.04 | 5.56x10^-7^ | 0.93 |
| Model adjusted for age, sex and principal components. Odds ratio and confidence interval reported for binary variables and Beta and standard error for continuous variables.  Chr=chromosome, SNP=single nucleotide polymorphism, BP=base pair, A1=reference allele, A2=alternative allele, MAF=minor allele frequency (reference allele), OR=odds ratio of A1, BETA= beta coefficient, 95 % CI = 95% Confidence Interval, SE = Standard Error, RSQ= Imputation Quality | | | | | | | | | | |


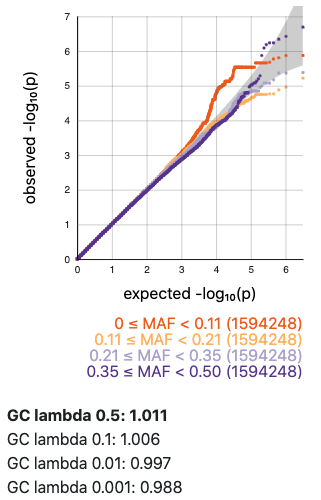


Figure S12. Regular cannabis use QQ Plot


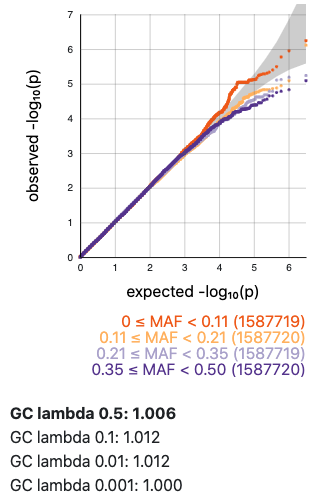


Figure S13. Heaviness of cannabis use QQ plot

| Table S3. SNPs and associated outcomes stratified by sex | | | | | | |
| --- | --- | --- | --- | --- | --- | --- |
| **Outcome** | **SNP** | **N** | **Reference Allele** | **OR/BETA** | **95 % CI/SE** | **P** |
| **Regular cannabis use** | **rs1813412** |  | G |  |  |  |
|  | Males | 1497 |  | 1.31 | 1.13, 1.52 | 4.32x10^-4^ |
|  | Females | 1109 |  | 1.47 | 1.23, 1.76 | 2.33x10^-5^ |
|  | Interaction | 2616 |  | 1.15 | 0.92, 1.46 | 0.23 |
| **Heaviness of cannabis use** | **rs62378502** |  | C |  |  |  |
|  | Males | 799 |  | 0.19 | 0.05 | 6.59x10^-5^ |
|  | Females | 490 |  | 0.19 | 0.07 | 3.44x10^-3^ |
|  | Interaction | 1293 |  | 0.01 | 0.08 | 0.92 |
| Model adjusted for age and principal components. Odds ratio and confidence interval reported for binary variables and Beta and standard error for continuous variables.  OR=odds ratio, BETA= beta coefficient, 95% CI = 95% confidence interval levels (lower, upper), SE=standard error | | | | | | |

| Table S4. PRS model fit across thresholds for each outcome for CUD | | | | | | |
| --- | --- | --- | --- | --- | --- | --- |
| **Outcome** | **Threshold** | **R^2^** | **Coefficient** | **SE** | **P** | **Number of SNPs** |
| **Regular cannabis use** | 5.0x10^-8^ | 5.11x10^-4^ | 1.6 | 1.6 | 0.31 | 2 |
|  | 1.0x10^-5^ | 3.82x10^-4^ | 7.1 | 8.0 | 0.38 | 45 |
|  | 0.0001 | 2.38x10^-3^ | 38.6 | 17.6 | **0.028** | 204 |
|  | 0.001 | 2.28x10^-3^ | 104.6 | 48.7 | **0.032** | 1280 |
|  | 0.05 | 4.20x10^-4^ | 277.3 | 300.3 | 0.36 | 36660 |
|  | 0.1 | 7.68x10^-4^ | 520.8 | 417.1 | 0.21 | 66179 |
|  | 0.2 | 2.67x10^-4^ | 436.7 | 593.0 | 0.46 | 118682 |
|  | 0.3 | 7.22x10^-5^ | 285.6 | 745.9 | 0.70 | 166435 |
|  | 0.4 | 1.79x10^-6^ | 53.7 | 889.5 | 0.95 | 209778 |
|  | 0.5 | 3.00x10^-5^ | 252.5 | 1023.3 | 0.81 | 249212 |
|  | 1 | 2.54x10^-5^ | 344.2 | 1514.9 | 0.82 | 381569 |
| **Heaviness of cannabis use** | 5.0x10^-8^ | 4.14x10^-4^ | 0.5 | 0.7 | 0.46 | 2 |
|  | 1.0x10^-5^ | 9.06x10^-4^ | -3.7 | 3.4 | 0.28 | 45 |
|  | 0.0001 | 7.30x10^-4^ | -7.3 | 7.5 | 0.33 | 203 |
|  | 0.001 | 6.35x10^-7^ | -0.6 | 20.8 | 0.98 | 1280 |
|  | 0.05 | 8.16x10^-5^ | 42.1 | 129.0 | 0.74 | 36662 |
|  | 0.1 | 5.51x10^-5^ | 48.3 | 180.0 | 0.79 | 66241 |
|  | 0.2 | 5.42x10^-5^ | 67.9 | 255.2 | 0.79 | 118664 |
|  | 0.3 | 3.96x10^-5^ | 73.1 | 321.4 | 0.82 | 166396 |
|  | 0.4 | 4.59x10^-5^ | -94.1 | 384.8 | 0.81 | 209735 |
|  | 0.5 | 3.44x10^-5^ | -94.0 | 443.4 | 0.83 | 249091 |
|  | 1 | 4.64x10^-5^ | -161.9 | 658.3 | 0.81 | 381358 |
| **MCQ-SF** | 5.0x10^-8^ | 1.35x10^-3^ | 0.3 | 0.27 | 0.29 | 2 |
|  | 1.0x10^-5^ | 4.01x10^-5^ | 0.2 | 1.3 | 0.85 | 45 |
|  | 0.0001 | 2.89x10^-4^ | 1.4 | 2.8 | 0.62 | 203 |
|  | 0.001 | 1.94x10^-4^ | 3.1 | 7.8 | 0.69 | 1285 |
|  | 0.05 | 2.15x10^-4^ | 20.5 | 48.3 | 0.67 | 36679 |
|  | 0.1 | 8.22x10^-5^ | 17.6 | 67.0 | 0.79 | 66169 |
|  | 0.2 | 1.30x10^-7^ | -1.0 | 95.7 | 0.99 | 118625 |
|  | 0.3 | 4.23x10^-5^ | 22.7 | 120.1 | 0.85 | 166251 |
|  | 0.4 | 9.06x10^-5^ | -39.9 | 144.4 | 0.78 | 209574 |
|  | 0.5 | 1.31x10^-4^ | -55.2 | 166.4 | 0.74 | 248834 |
|  | 1 | 8.07x10^-5^ | -64.4 | 247.2 | 0.79 | 380998 |
| Model adjusted for age, sex and principal components.  R^2^=Variance explained by the PRS, SE=standard error, Number of SNPs=number of SNPs included in the PRS, FDR=False Discovery Rate | | | | | | |
| \| Table S5. PRS model fit across thresholds for each outcome for lifetime cannabis use \| \| \| \| \| \| \| \| \| --- \| --- \| --- \| --- \| --- \| --- \| --- \| --- \| \| **Outcome** \| **Threshold** \| **R^2^** \| **Coefficient** \| **SE** \| **P** \| **Number of SNPs** \| **FDR corrected p-value** \| \| **Regular cannabis use** \| 5.0x10^-8^ \| 1.89x10^-3^ \| 17.3 \| 3.7 \| 0.05 \| 9 \| 0.27 \| \| 1.0x10^-5^ \| 2.76x10^-3^ \| 32.8 \| 13.9 \| **0.02** \| 74 \| 0.20 \| \| 0.0001 \| 1.48x10^-3^ \| 58.7 \| 33.9 \| 0.08 \| 328 \| 0.30 \| \| 0.001 \| 8.68x10^-5^ \| 27.9 \| 66.4 \| 0.67 \| 1695 \| 0.74 \| \| 0.05 \| 5.44x10^-5^ \| 107.2 \| 322.8 \| 0.74 \| 38144 \| 0.74 \| \| 0.1 \| 7.29x10^-4^ \| 533.6 \| 438.7 \| 0.22 \| 67766 \| 0.49 \| \|  \| 0.2 \| 7.47x10^-4^ \| 763.8 \| 620.4 \| 0.22 \| 119460 \| 0.49 \| \|  \| 0.3 \| 2.69x10^-4^ \| 573.3 \| 776.4 \| 0.46 \| 164811 \| 0.72 \| \|  \| 0.4 \| 3.22x10^-4^ \| 747.5 \| 924.9 \| 0.42 \| 206272 \| 0.72 \| \|  \| 0.5 \| 1.34x10^-4^ \| 554.4 \| 1064.0 \| 0.60 \| 244257 \| 0.74 \| \|  \| 1 \| 1.76x10^-4^ \| 951.6 \| 1590.0 \| 0.55 \| 376270 \| 0.74 \| \| **Heaviness of cannabis use** \| 5.0x10^-8^ \| 2.50x10^-5^ \| -0.3 \| 1.6 \| 0.86 \| 9 \| 0.86 \| \| 1.0x10^-5^ \| 1.18x10^-3^ \| -7.5 \| 6.0 \| 0.21 \| 74 \| 0.48 \| \| 0.0001 \| 1.54x10^-3^ \| -20.9 \| 14.7 \| 0.16 \| 325 \| 0.48 \| \| 0.001 \| 1.18x10^-3^ \| -36.3 \| 29.3 \| 0.22 \| 1692 \| 0.48 \| \| 0.05 \| 6.75x10^-4^ \| -131.3 \| 139.8 \| 0.35 \| 38125 \| 0.51 \| \| 0.1 \| 3.25x10^-4^ \| -124.1 \| 190.6 \| 0.52 \| 67732 \| 0.57 \| \| 0.2 \| 5.42x10^-4^ \| -227.9 \| 271.0 \| 0.40 \| 119435 \| 0.51 \| \| 0.3 \| 4.97x10^-4^ \| -273.2 \| 339.2 \| 0.42 \| 164791 \| 0.51 \| \| 0.4 \| 8.64x10^-4^ \| -429.6 \| 404.6 \| 0.29 \| 206217 \| 0.51 \| \| 0.5 \| 1.17x10^-3^ \| -574.5 \| 465.4 \| 0.22 \| 244152 \| 0.48 \| \| 1 \| 1.82x10^-3^ \| -1072.5 \| 695.0 \| 0.12 \| 376062 \| 0.48 \| \| **MCQ-SF** \| 5.0x10^-8^ \| 3.30x10^-3^ \| 1.0 \| 0.6 \| 0.10 \| 9 \| 0.27 \| \| 1.0x10^-5^ \| 1.02x10^-2^ \| 6.8 \| 2.3 \| **3.39x10^-3^** \| 74 \| **0.04** \| \| 0.0001 \| 4.72x10^-3^ \| 11.2 \| 5.6 \| 0.05 \| 326 \| 0.25 \| \| 0.001 \| 3.23x10^-3^ \| 18.5 \| 11.2 \| 0.10 \| 1688 \| 0.27 \| \| 0.05 \| 2.35x10^-4^ \| -23.3 \| 52.3 \| 0.66 \| 38109 \| 0.97 \| \| 0.1 \| 2.57x10^-4^ \| -32.6 \| 70.0 \| 0.64 \| 67710 \| 0.97 \| \| 0.2 \| 1.74x10^-4^ \| -38.2 \| 99.9 \| 0.70 \| 119409 \| 0.97 \| \| 0.3 \| 2.69x10^-5^ \| -18.9 \| 125.7 \| 0.88 \| 164681 \| 0.97 \| \| 0.4 \| 4.13x10^-8^ \| 0.9 \| 150.7 \| 1.00 \| 206083 \| 1.00 \| \| 0.5 \| 6.80x10^-5^ \| -41.5 \| 173.7 \| 0.81 \| 243927 \| 0.97 \| \| 1 \| 3.65x10^-5^ \| -45.6 \| 260.4 \| 0.86 \| 375635 \| 0.97 \| \| Model adjusted for age, sex and principal components.  R^2^=Variance explained by the PRS, SE=standard error, Number of SNPs=number of SNPs included in the PRS, FDR=False Discovery Rate \| \| \| \| \| \| \|  \| | | | | | | |
|  | | | | | | |
|  | | | | | | |


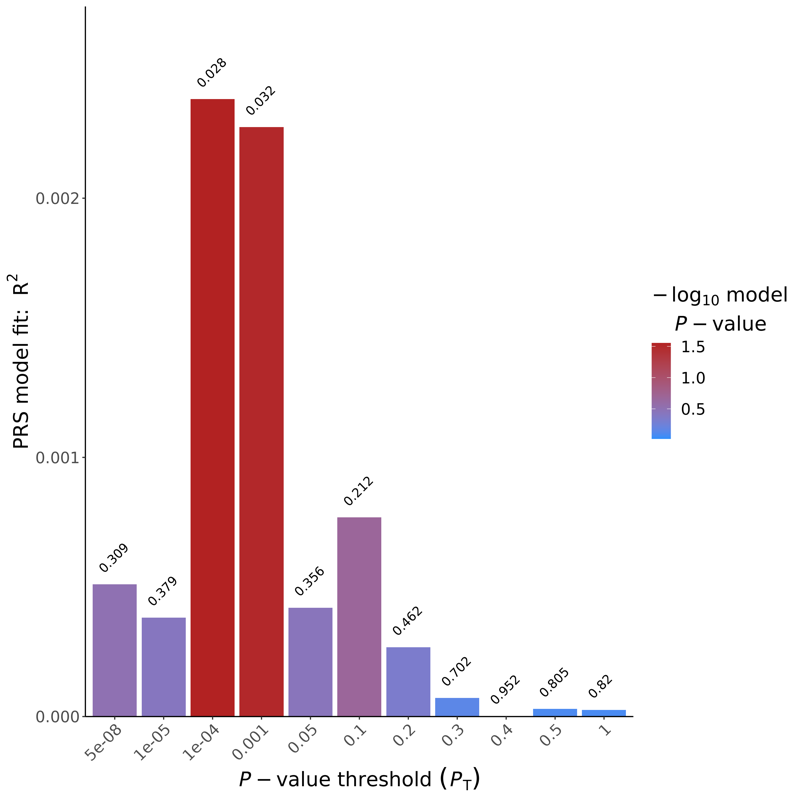


Figure S14. Regular cannabis PRS model fit generated by PRSice-2 using CUD GWAS summary statistics(19)


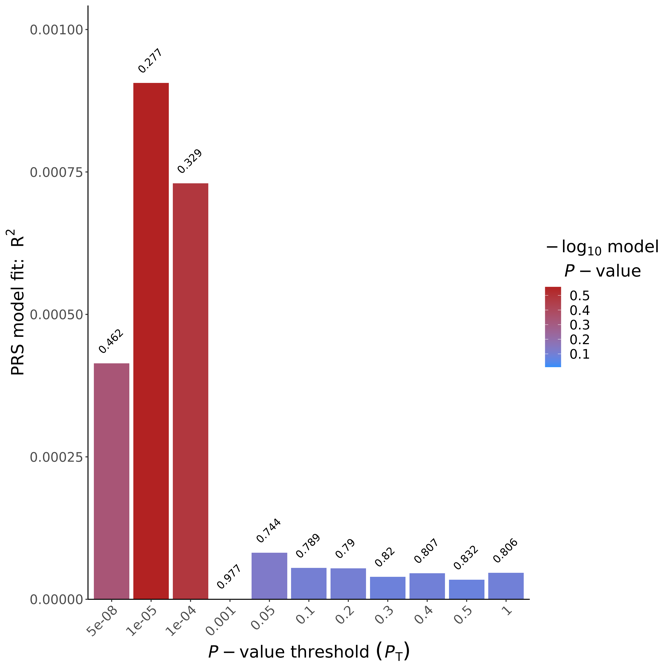


Figure S15. Heaviness of use PRS model fit generated by PRSice-2 using CUD GWAS summary statistics (19)


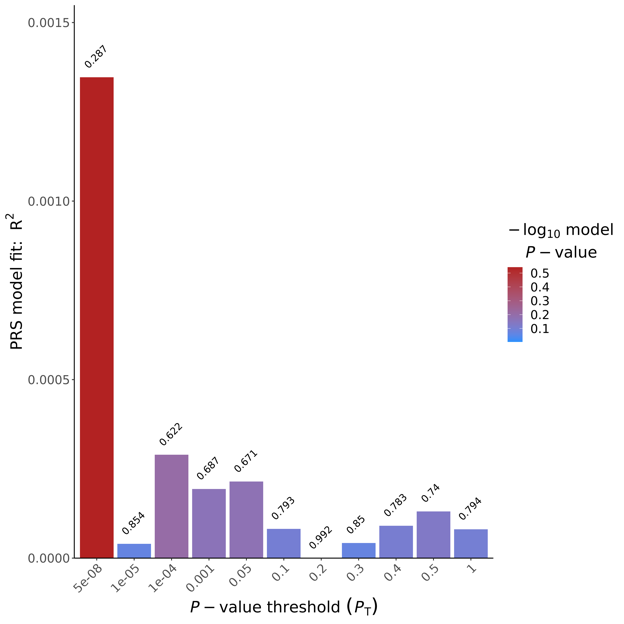


Figure S16. Cannabis cravings PRS model of fit generated by PRSice-2 using CUD GWAS summary statistics (19)


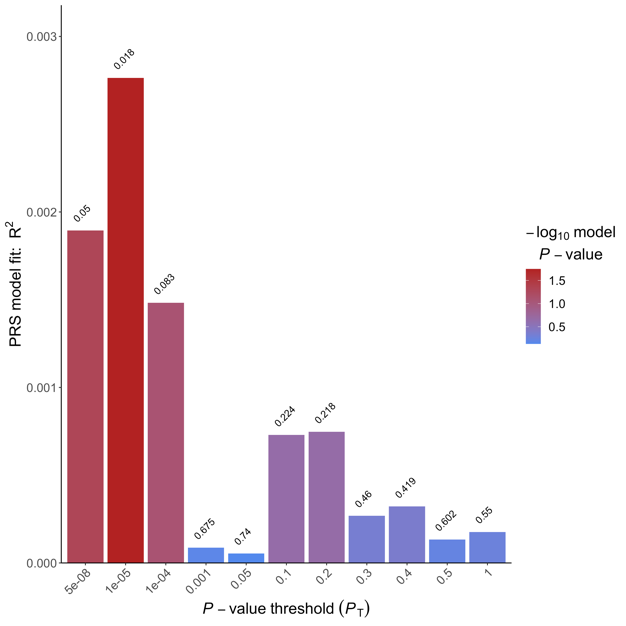


Figure S17. Regular cannabis use PRS model of fit generated by PRSice-2 using lifetimes cannabis use GWAS summary statistics (19)


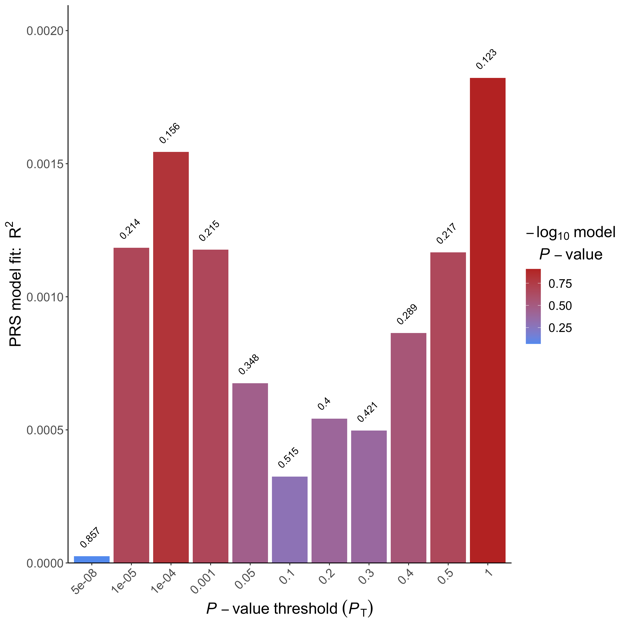


Figure S18. Heaviness of cannabis use PRS model of fit generated by PRSice-2 using lifetime cannabis use GWAS summary statistics (19)


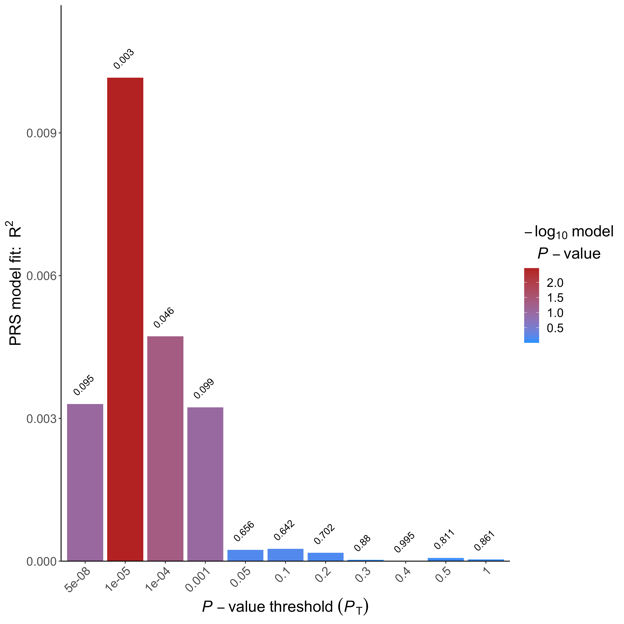


Figure S19. Cannabis cravings PRS model of fit generated by PRSice-2 using lifetimes cannabis use GWAS summary statistics (19)

| Table S6. Results from PhenoScanner for rs1813412(22,23) | | | | | | | |
| --- | --- | --- | --- | --- | --- | --- | --- |
| **rsid** | **Position (Chr:BP)** | **A1/A2** | **Trait** | **PMID** | **BETA** | **P** | **N** |
| **rs1813412** | chr17:22193901 | A/G | Acute pharyngitis | UKBB | -0.0002371 | 1.69x10^-4^ | 337199 |
| **rs1813412** | chr17:22193901 | A/G | Cause of death: other interstitial pulmonary diseases with fibrosis | UKBB | -0.007057 | 1.74x10^-4^ | 7637 |
| **rs1813412** | chr17:22193901 | A/G | Follicular cysts of skin and subcutaneous tissue | UKBB | 0.0003778 | 3.69x10^-4^ | 337159 |
| **rs1813412** | chr17:22193901 | A/G | Maternal smoking around birth | UKBB | 0.004399 | 4.73x10^-4^ | 289727 |
| **rs1813412** | chr17:22193901 | A/G | Self-reported back pain | UKBB | -0.001074 | 4.92x10^-4^ | 337199 |
| **rs1813412** | chr17:22193901 | A/G | Self-reported prostate problem | UKBB | 0.0006226 | 7.56x10-4 | 337159 |
| Chr=chromosome, BP=base pair, A1=reference allele, A2=alternative allele, BETA= beta coefficient, P= p-value, N= sample size | | | | | | | |

Reporting checklist for genetic association study.

Based on the STREGA guidelines.

**Instructions to authors**

Complete this checklist by entering the page numbers from your manuscript where readers will find each of the items listed below.

Your article may not currently address all the items on the checklist. Please modify your text to include the missing information. If you are certain that an item does not apply, please write "n/a" and provide a short explanation.

Upload your completed checklist as an extra file when you submit to a journal.

In your methods section, say that you used the STREGAreporting guidelines, and cite them as:

Little J, Higgins JP, Ioannidis JP, Moher D, Gagnon F, von Elm E, Khoury MJ, Cohen B, Davey-Smith G, Grimshaw J, Scheet P, Gwinn M, Williamson RE, Zou GY, Hutchings K, Johnson CY, Tait V, Wiens M, Golding J, van Duijn C, McLaughlin J, Paterson A, Wells G, Fortier I, Freedman M, Zecevic M, King R, Infante-Rivard C, Stewart A, Birkett N; STrengthening the REporting of Genetic Association Studies. STrengthening the REporting of Genetic Association Studies (STREGA): An Extension of the STROBE Statement.

|  |  | Reporting Item | Page Number |
| --- | --- | --- | --- |
| **Title and abstract** |  |  | 1-4 |
| Title | [#1a](https://www.goodreports.org/strega/info/#1a) | Indicate the study’s design with a commonly used term in the title or the abstract |  |
| Abstract | [#1b](https://www.goodreports.org/strega/info/#1b) | Provide in the abstract an informative and balanced summary of what was done and what was found |  |
| **Background/rationale** |  |  | 5-6 |
|  | [#2](https://www.goodreports.org/strega/info/#2) | Explain the scientific background and rationale for the investigation being reported |  |
| **Objectives** |  |  | 6-7 |
|  | [#3](https://www.goodreports.org/strega/info/#3) | State specific objectives, including any prespecified hypotheses. State if the study is the first report of a genetic association, a replication effort, or both. |  |
| **Study design** |  |  | 7-8 |
|  | [#4](https://www.goodreports.org/strega/info/#4) | Present key elements of study design early in the paper |  |
| **Setting** |  |  | 7-8 |
|  | [#5](https://www.goodreports.org/strega/info/#5) | Describe the setting, locations, and relevant dates, including periods of recruitment, exposure, follow-up, and data collection |  |
| **Eligibility criteria** |  |  | 8 |
|  | [#6a](https://www.goodreports.org/strega/info/#6a) | Cohort study – Give the eligibility criteria, and the sources and methods of selection of participants. Describe methods of follow-up. Case-control study – Give the eligibility criteria, and the sources and methods of case ascertainment and control selection. Give the rationale for the choice of cases and controls. Cross-sectional study – Give the eligibility criteria, and the sources and methods of selection of participants. Give information on the criteria and methods for selection of subsets of participants from a larger study, when relevant. |  |
|  | [#6b](https://www.goodreports.org/strega/info/#6b) | Cohort study – For matched studies, give matching criteria and number of exposed and unexposed. Case-control study – For matched studies, give matching criteria and the number of controls per case. |  |
| **Variables** |  |  | 8-9 |
|  | [#7a](https://www.goodreports.org/strega/info/#7a) | Clearly define all outcomes, exposures, predictors, potential confounders, and effect modifiers. Give diagnostic criteria, if applicable |  |
|  | [#7b](https://www.goodreports.org/strega/info/#7b) | Clearly define genetic exposures (genetic variants) using a widely-used nomenclature system. Identify variables likely to be associated with population stratification (confounding by ethnic origin). |  |
| **Data sources/measurement** |  |  | 9-11  Supplementary File 1 |
|  | [#8a](https://www.goodreports.org/strega/info/#8a) | For each variable of interest give sources of data and details of methods of assessment (measurement). Describe comparability of assessment methods if there is more than one group. Give information separately for for exposed and unexposed groups if applicable. |  |
|  | [#8b](https://www.goodreports.org/strega/info/#8b) | Describe laboratory methods, including source and storage of DNA, genotyping methods and platforms (including the allele calling algorithm used, and its version), error rates and call rates. State the laboratory / centre where genotyping was done. Describe comparability of laboratory methods if there is more than one group. Specify whether genotypes were assigned using all of the data from the study simultaneously or in smaller batches. |  |
| **Bias** |  |  | 20 |
|  | [#9a](https://www.goodreports.org/strega/info/#9a) | Describe any efforts to address potential sources of bias |  |
|  | [#9b](https://www.goodreports.org/strega/info/#9b) | Describe any efforts to address potential sources of bias |  |
| **Study size** |  |  | 10-11 |
|  | [#10](https://www.goodreports.org/strega/info/#10) | Explain how the study size was arrived at |  |
| **Quantitative variables** |  |  | 8-11 |
|  | [#11](https://www.goodreports.org/strega/info/#11) | Explain how quantitative variables were handled in the analyses. If applicable, describe which groupings were chosen, and why. If applicable, describe how effects of treatment were dealt with. |  |
| **Statistical methods** |  |  | 11  Supplementary File 1 |
|  | [#12a](https://www.goodreports.org/strega/info/#12a) | Describe all statistical methods, including those used to control for confounding. State software version used and options (or settings) chosen. |  |
|  | [#12b](https://www.goodreports.org/strega/info/#12b) | Describe any methods used to examine subgroups and interactions |  |
|  | [#12c](https://www.goodreports.org/strega/info/#12c) | Explain how missing data were addressed |  |
|  | [#12d](https://www.goodreports.org/strega/info/#12d) | If applicable, explain how loss to follow-up was addressed |  |
|  | [#12e](https://www.goodreports.org/strega/info/#12e) | Describe any sensitivity analyses |  |
|  | [#12f](https://www.goodreports.org/strega/info/#12f) | State whether Hardy-Weinberg equilibrium was considered and, if so, how. |  |
|  | [#12g](https://www.goodreports.org/strega/info/#12g) | Describe any methods used for inferring genotypes or haplotypes |  |
|  | [#12h](https://www.goodreports.org/strega/info/#12h) | Describe any methods used to assess or address population stratification. |  |
|  | [#12i](https://www.goodreports.org/strega/info/#12i) | Describe any methods used to address multiple comparisons or to control risk of false positive findings. |  |
|  | [#12j](https://www.goodreports.org/strega/info/#12j) | Describe any methods used to address and correct for relatedness among subjects |  |
| **Participants** |  |  | 12-13 |
|  | [#13a](https://www.goodreports.org/strega/info/#13a) | Report numbers of individuals at each stage of study—eg numbers potentially eligible, examined for eligibility, confirmed eligible, included in the study, completing follow-up, and analysed. Give information separately for for exposed and unexposed groups if applicable. Report numbers of individuals in whom genotyping was attempted and numbers of individuals in whom genotyping was successful. |  |
|  | [#13b](https://www.goodreports.org/strega/info/#13b) | Give reasons for non-participation at each stage |  |
|  | [#13c](https://www.goodreports.org/strega/info/#13c) | Consider use of a flow diagram |  |
| **Descriptive data** |  |  | 13-14 |
|  | [#14a](https://www.goodreports.org/strega/info/#14a) | Give characteristics of study participants (eg demographic, clinical, social) and information on exposures and potential confounders. Give information separately for exposed and unexposed groups if applicable. Consider giving information by genotype |  |
|  | [#14b](https://www.goodreports.org/strega/info/#14b) | Indicate number of participants with missing data for each variable of interest |  |
|  | [#14c](https://www.goodreports.org/strega/info/#14c) | Cohort study – Summarize follow-up time, e.g. average and total amount. |  |
| **Outcome data** |  |  | 14-17 |
|  | [#15](https://www.goodreports.org/strega/info/#15) | Cohort study Report numbers of outcome events or summary measures over time.Give information separately for exposed and unexposed groups if applicable. Report outcomes (phenotypes) for each genotype category over time Case-control study – Report numbers in each exposure category, or summary measures of exposure.Give information separately for cases and controls . Report numbers in each genotype category. Cross-sectional study – Report numbers of outcome events or summary measures. Give information separately for exposed and unexposed groups if applicable. Report outcomes (phenotypes) for each genotype category |  |
| **Main results** |  |  | 14-17 |
|  | [#16a](https://www.goodreports.org/strega/info/#16a) | Give unadjusted estimates and, if applicable, confounder-adjusted estimates and their precision (eg, 95% confidence interval). Make clear which confounders were adjusted for and why they were included |  |
|  | [#16b](https://www.goodreports.org/strega/info/#16b) | Report category boundaries when continuous variables were categorized |  |
|  | [#16c](https://www.goodreports.org/strega/info/#16c) | If relevant, consider translating estimates of relative risk into absolute risk for a meaningful time period |  |
|  | [#16d](https://www.goodreports.org/strega/info/#16d) | Report results of any adjustments for multiple comparisons |  |
| **Other analyses** |  |  | 14-17 |
|  | [#17a](https://www.goodreports.org/strega/info/#17a) | Report other analyses done—e.g., analyses of subgroups and interactions, and sensitivity analyses |  |
|  | [#17b](https://www.goodreports.org/strega/info/#17b) | Report other analyses done—e.g., analyses of subgroups and interactions, and sensitivity analyses |  |
|  | [#17c](https://www.goodreports.org/strega/info/#17c) | Report other analyses done—e.g., analyses of subgroups and interactions, and sensitivity analyses |  |
| **Key results** |  |  | 17-19 |
|  | [#18](https://www.goodreports.org/strega/info/#18) | Summarise key results with reference to study objectives |  |
| **Limitations** |  |  | 19-21 |
|  | [#19](https://www.goodreports.org/strega/info/#19) | Discuss limitations of the study, taking into account sources of potential bias or imprecision. Discuss both direction and magnitude of any potential bias. |  |
| **Interpretation** |  |  | 17-19 |
|  | [#20](https://www.goodreports.org/strega/info/#20) | Give a cautious overall interpretation considering objectives, limitations, multiplicity of analyses, results from similar studies, and other relevant evidence. |  |
| **Generalisability** |  |  | 20-21 |
|  | [#21](https://www.goodreports.org/strega/info/#21) | Discuss the generalisability (external validity) of the study results |  |
| **Funding** |  |  | 21 |
|  | [#22](https://www.goodreports.org/strega/info/#22) | Give the source of funding and the role of the funders for the present study and, if applicable, for the original study on which the present article is based |  |

None The STREGA checklist is distributed under the terms of the Creative Commons Attribution License CC-BY. This checklist can be completed online using <https://www.goodreports.org/>, a tool made by the [EQUATOR Network](https://www.equator-network.org) in collaboration with [Penelope.ai](https://www.penelope.ai)

References

1. Samaan Z, Bawor M, Dennis BB, Plater C, Varenbut M, Daiter J, et al. Genetic influence on methadone treatment outcomes in patients undergoing methadone maintenance treatment for opioid addiction: a pilot study. Neuropsychiatr Dis Treat. 2014;1503–8.

2. Purcell S, Neale B, Todd-Brown K, Thomas L, Ferreira MAR, Bender D, et al. PLINK: a tool set for whole-genome association and population-based linkage analyses. Am J Hum Genet. 2007;81(3):559–75.

3. RStudio Team. RStudio: Integrated Development for R. Boston, MA: RStudio; 2020.

4. R Development Core Team. R: A Language and Environment for Statistical Computing. 2011.

5. Das S, Forer L, Schönherr S, Sidore C, Locke AE, Kwong A, et al. Next-generation genotype imputation service and methods. Nat Genet. 2016;48(10):1284–7.

6. DANgenotek. ORAgene-DISCOVER - OGR-500 [Internet]. 2022. Available from: https://www.dnagenotek.com/us/products/collection-human/oragene-discover/500-series/OGR-500.html

7. Centre d’expertise et de services Génome Québec. DNA Extraction Servics [Internet]. 2020. Available from: https://static1.squarespace.com/static/5a53cd127131a5135e2456ba/t/5f63f155343fba7cda512035/1600385365852/UserGuide_DNAExtraction_en.pdf

8. Centre d’expertise et de services Génome Québec [Internet]. Available from: https://cesgq.com/

9. illumina. Infinium Global Screening Array - 24 v1.0 [Internet]. 2017. Available from: https://grcf.jhmi.edu/wp-content/uploads/2017/12/infinium-commercial-gsa-data-sheet-370-2016-016.pdf

10. illumina. GenomeStudio [Internet]. 2022. Available from: https://support.illumina.com/array/array_software/genomestudio/downloads.html

11. illumina. Global Screening Array Manifest File Release Notes. 2018.

12. Taliun D, Harris DN, Kessler MD, Carlson J, Szpiech ZA, Torres R, et al. Sequencing of 53,831 diverse genomes from the NHLBI TOPMed Program. Nature. 2021;590(7845):290–9.

13. Rayner W. McCarthy Tools.

14. Loh P-R, Danecek P, Palamara PF, Fuchsberger C, Reshef YA, Finucane HK, et al. Reference-based phasing using the Haplotype Reference Consortium panel. Nat Genet. 2016;48(11):1443.

15. TOPMed. TOPMed Imputation Server - Reference Panels [Internet]. Available from: https://topmedimpute.readthedocs.io/en/latest/reference-panels.html

16. Google Cloud. Google Cloud. 2022.

17. Bycroft C, Freeman C, Petkova D, Band G, Elliott LT, Sharp K, et al. The UK Biobank resource with deep phenotyping and genomic data. Nature. 2018;562(7726):203–9.

18. Johnson EC, Demontis D, Thorgeirsson TE, Walters RK, Polimanti R, Hatoum AS, et al. A large-scale genome-wide association study meta-analysis of cannabis use disorder. The Lancet Psychiatry. 2020;7(12):1032–45.

19. Choi SW, O’Reilly PF. PRSice-2: Polygenic Risk Score software for biobank-scale data. Gigascience. 2019;8(7):giz082.

20. Choi SW, Mak TS-H, O’Reilly PF. Tutorial: a guide to performing polygenic risk score analyses. Nat Protoc. 2020;15(9):2759–72.

21. Dennis BB, Sanger N, Bawor M, Naji L, Plater C, Worster A, et al. A call for consensus in defining efficacy in clinical trials for opioid addiction: combined results from a systematic review and qualitative study in patients receiving pharmacological assisted therapy for opioid use disorder. Trials. 2020;21(1):1–16.

22. Staley JR, Blackshaw J, Kamat MA, Ellis S, Surendran P, Sun BB, et al. PhenoScanner: a database of human genotype–phenotype associations. Bioinformatics. 2016;32(20):3207–9.

23. Kamat MA, Blackshaw JA, Young R, Surendran P, Burgess S, Danesh J, et al. PhenoScanner V2: an expanded tool for searching human genotype–phenotype associations. Bioinformatics. 2019;35(22):4851–3.
